# SCULPT: Medical student and resident doctor comprehension, uptake of learning and perception of aesthetic surgery and training

**DOI:** 10.1016/j.jpra.2026.03.043

**Published:** 2026-04-04

**Authors:** Anuska Shah, Rananjay Singh, Cheuk Ying Kyleen Kiew, Umar Rehman, Daniella Soussi, Anna Chiara Corriero, Zahra Abdali, Krisada Ooi, Mohammad Alradhawi, Mohammed Sohaib Sarwar, Rakesh Anand, Peter A. Brennan, Simon Eccles, Theodore Pezas, UKPRC Collaborators, Tharsiga Yogarajah, Shreya Kantamneni, Samaika Jha, Maurika Vathanan, Gwennan Shewring, Jade Miller, Yun Mie Mie Latt, Archana Pradeep, Raveenjot Nagra, Yiu Ka Nathaniel Leung, Jobina Antony, Aksha Patel, Wiktoria Karbowniczek, Bethany Hatten, Reginold Baskaran, Owen Conlan, Oladimeji John Abiodun, Jonathan Herron, Ciaran Sandhu, Anisha Jain, Mehreen Ahmad, Hari McGrath, Ahmed Turkman, Ananya Shearman, Natalia Makhdoom, Subham Roy, Soyoung Lee, Shems Almusawi, Lawson Faulkner, Shalayna Sakaria, Mohamed Alaktaa, Luanne Lai, Krzysztof Sosnowski, Krishna Keshav Kulkarni, Hajar Abdulla, Gareth Goh, Asta Kirkham, Abbie Carter, Khushi Chhabra, Aaliyah Bizinga, Sancia Fernando, Louise Leijonberg

**Affiliations:** a Imperial College London, London, United Kingdom; b UCL Division of Surgery and Interventional Science, University College London, London, United Kingdom; c Bone and Joint Health, Blizard Institute, Queen Mary University London, United Kingdom; d Royal Free London NHS Foundation Trust, London, United Kingdom; e NHS Lothian, Edinburgh, United Kingdom; f University of Aberdeen, Aberdeen, United Kingdom; g University of Edinburgh, Edinburgh, United Kingdom; h Department of Oral and Maxillofacial Surgery, Northwick Park Hospital, London, United Kingdom; i Department of Oral and Maxillofacial Surgery, Bradford Teaching Hospitals NHS Foundation Trust, Yorkshire, United Kingdom; j Department of Dermatology, Guy’s and St Thomas’ NHS Foundation Trust, London, United Kingdom; k Department of Maxillofacial Surgery, Queen Alexandra Hospital, Portsmouth, United Kingdom; l Department of Plastic Surgery, Great Ormond Street Hospital for Children NHS Foundation Trust, London, United Kingdom; m Evelina London Cleft Service, Guy’s and St Thomas’ NHS Foundation Trust, London, United Kingdom; n Faculty of Medical Sciences, University College London, London, United Kingdom

**Keywords:** Aesthetic surgery, Medical education, Surgical training, Non-surgical aesthetics, Regulation, Patient safety

## Abstract

**Background:**

As demand for aesthetic procedures continues to grow in the UK, alongside presentations of aesthetic-related complications, understanding current training provision and clinician preparedness is essential to inform educational curriculum development and regulatory policy.

**Methods:**

A cross-sectional online survey was conducted across UK medical schools and postgraduate medical education regions to evaluate UK medical students’ and resident doctors’ exposure to, knowledge of, and perceptions of aesthetic practice.

**Results:**

A total of 2369 participants completed the survey (1757 students; 612 residents). Formal aesthetic teaching was uncommon: 10.9% of students (*n* = 191) and 10.5% of resident doctors (*n* = 64) received undergraduate teaching, and 11.9% of residents (*n* = 73) had postgraduate exposure. Self-reported knowledge scores ranged from 1.72 to 2.65/5 for surgical procedures and 2.32–2.99/5 for non-surgical procedures. However, 40.7% of residents (*n* = 249) had already managed at least one aesthetic complication, with 78.3% (*n* = 195) arising from procedures performed abroad. Career interest was substantial (38.2% overall), with participants identifying consent principles (4.08 ± 1.02) and procedure overview (4.00 ± 1.00) as priority curriculum additions. Social media was the most influential source shaping perceptions (residents 3.64 ± 1.17; students 3.51 ± 1.30), while formal education had minimal impact. Strong consensus emerged on regulatory needs: 86.4% of residents and 78.6% of students identified lack of regulation in the non-surgical sector as a significant concern, with 88.2% and 86.0% respectively supporting restriction of invasive non-surgical procedures to medically trained professionals.

**Conclusion:**

These findings provide an evidence base for developing structured aesthetic curricula and strengthening regulatory frameworks to better prepare clinicians for this expanding area of practice.

## Introduction

Aesthetic surgery encompasses procedures performed to enhance physical appearance, including surgical interventions such as rhinoplasty and liposuction, as well as non-surgical treatments such as botulinum toxin and dermal fillers.^1^ In the United Kingdom (UK), the General Medical Council (GMC) stipulates that only doctors on the Specialist Register should perform surgical aesthetic procedures. The Royal College of Surgeons (RCS) offers optional certification pathways; however, there are no statutory training requirements for non-surgical aesthetics.^2^^,^^3^ Up to 31% of UK aesthetic practitioners are not licensed healthcare professionals,^4^ highlighting the need for clearer regulatory frameworks to improve patient safety. Against this backdrop, the British Association of Aesthetic Plastic Surgeons (BAAPS) represents several hundred plastic surgeons,^5^ while the Joint Council for Cosmetic Practitioners registers just over one thousand practitioners,^6^ these organizations collectively represent only a subset of the wider UK aesthetic workforce, which has been estimated at approximately 7800 practitioners and may be considerably larger, with some studies suggesting up to 20,000 individuals performing non-surgical procedures.^7^^,^^8^ This disparity highlights the urgent need to broaden regulatory engagement to include practitioners across specialties and professional backgrounds, ensuring more comprehensive oversight of the aesthetic sector.

Demand for aesthetic procedures has grown substantially over the past decade.^9^ In 2024, BAAPS documented 27,462 cosmetic procedures, with breast augmentation, breast reduction, and blepharoplasty most commonly performed.^10^ Growth has been particularly notable in the non-surgical sector, with approximately 900,000 botulinum toxin and 370,000 dermal filler procedures performed annually, largely in private practice.^4^ Social media platforms, particularly TikTok, have increased public awareness and interest in aesthetic treatments.^11^ The increasing use of glucagon-like peptide-1 weight loss medications has coincided with rising interest in injectable fillers, skin tightening, and body contouring.^12^ This evolving landscape has facilitated patient access to services based on cost and convenience, frequently without awareness of practitioner credentials or registration status.^13^

The increase in aesthetic procedures has been associated with a rise in complications, including venous thromboembolism, infection, skin necrosis, and blindness, all of which require prompt recognition and management.^14^^,^^15^ Some complications presenting to the NHS arise from cosmetic tourism, where patients undergo procedures abroad and present after returning to the UK.^16^^,^^17^ Understanding these presentations is vital to ensuring clinicians across training stages are prepared to recognize and manage aesthetic-related complications.

Undergraduate medical curricula typically offer limited formal teaching in aesthetics^18^ whilst postgraduate training pathways vary in accessibility and structure.^19^ Both areas represent clear targets for evidence-based educational and regulatory reform. Developing structured training frameworks alongside rigorous professional standards could enhance clinician preparedness, support patient safety, and better align healthcare education with the dynamic nature of aesthetic practice.

The Medical Student and Resident Doctor Comprehension, Uptake of Learning and Perception of Aesthetic Surgery and Training (SCULPT) study evaluates medical students’ and resident doctors’ exposure to aesthetic medicine, identifies barriers to career engagement, and assesses attitudes toward the regulation and reputation of the aesthetic sector. Our findings aim to inform future curricular development and regulatory policy within the UK.

## Methods

### Study design

The SCULPT study was a national, cross-sectional survey of UK medical students and resident doctors. The study is reported in accordance with the STROBE (Strengthening the Reporting of Observational Studies in Epidemiology) guidelines.^20^

### Setting and participants

The survey was disseminated via Qualtrics XM (Provo, UT) between 08/10/2025 and 15/11/2025. Participants were recruited using non-random, convenience sampling through the UK Plastics Research Collaborative (UKPRC), with regional collaborators disseminating the survey to student societies and postgraduate training networks via social media. This approach achieved broad geographical representation across UK medical schools and postgraduate training regions; however, owing to this dissemination method, a formal response rate could not be calculated as the total number of individuals reached was not determined. This model has been adopted in prior published literature.^21^, ^22^, ^23^

Eligible participants were UK-based medical students (enrolled in a GMC-registered medical school) and resident doctors (undertaking a postgraduate training program or equivalent pathway within the NHS). Exclusion criteria were non-UK medical students and doctors not registered with the General Medical Council.

### Ethical approval

Ethical approval was granted by the Imperial College Education Ethics Review Process (EERP2425-128, 28/07/2025). Participation was voluntary, with electronic informed consent obtained. The information sheet outlined confidentiality and right to withdraw before submission. No identifiable data were collected and responses were accessible only to the lead researchers. Data storage complied with UK General Data Protection Regulation (GDPR) and the Declaration of Helsinki.

### Survey development

The questionnaire was designed based on a scoping review of studies extracted from PubMed, Embase, and Scopus to identify key themes and gaps within aesthetic surgery and medicine training. (See Supplementary Appendix A for full protocol.)

An initial draft underwent two iterative Delphi consensus cycles involving senior authors with expertise in aesthetics and medical education to establish content validity and ensure domain coverage. The revised instrument was then pilot tested with 10 medical students and 5 resident doctors to evaluate item clarity, face validity, and platform navigation, with further refinement based on feedback prior to final distribution.

The finalized survey (see Supplementary Appendix B) included five-point Likert scales, multiple-choice questions, and free-text responses. It was structured into six domains: (1) participant role, (2) demographics and experience, (3) knowledge of procedures, (4) perceptions of aesthetics, (5) barriers, ethics, and media influence, and (6) career interest, training pathways and curriculum integration.

### Study outcomes

The primary outcome of the SCULPT study was self-reported knowledge and perception of aesthetics among UK medical students and resident doctors. Secondary outcomes included reported barriers to aesthetic training, views on ethical and regulatory oversight, and interest in pursuing aesthetics as a career.

### Sample size

The target population consisted of an estimated 48,000 medical students enrolled at UK medical schools and 75,000 resident doctors in UK training programmes.^24^^,^^25^ A minimum sample size of 387 students and 382 residents was calculated using the Raosoft® sample size calculator (95% confidence level, 5% margin of error).

### Statistical methods

Data was managed in Microsoft Excel (Version 16.77.1) and analyzed using R (Version 4.0.0). Duplicate entries (identified by matching IP address and metadata) were removed prior to analysis. Incomplete submissions were automatically excluded by the Qualtrics platform, ensuring only fully completed surveys were included. Internal consistency of Likert-scale domains demonstrated high reliability (Cronbach’s alpha = 0.92). Descriptive statistics summarized participant demographics and survey responses. Categorical variables were reported as frequencies and percentages and continuous variables as means and standard deviations.

## Results

### Participant demographics

A total of 2369 participants completed the survey, comprising 1757 (74.2%) medical students from 44 UK medical schools and 612 (25.8%) resident doctors. Breakdown by medical student year, resident doctor training grade and gender and ethnicity distributions are provided in Table 1, Table 2, respectively. Geographical breakdowns and distribution are further highlighted in Supplementary Table 1 and Supplementary Figure 1.

**Table 1:** Year-of-study distribution for medical student participants and training grade distribution for resident doctors.

	Year of study/Training stage	*n* (%)
Graduate entry medical student	Year 1	48 (2.7%)
	Year 2	47 (2.7%)
	Year 3	42 (2.4%)
	Year 4	66 (3.8%)
	Intercalating	2 (0.1%)
Undergraduate entry medical student	Year 1	252 (14.3%)
	Year 2	214 (12.2%)
	Year 3	381 (21.7%)
	Year 4	291 (16.6%)
	Year 5	330 (18.8%)
	Intercalating	84 (4.8%)
Resident doctor	Foundation year 1 (FY1)	109 (17.8%)
	Foundation year 2 (FY2)	112 (18.3%)
	Core trainee (CT1–CT3)	135 (22.1%)
	Internal Medicine Training (IMT)	34 (5.6%)
	GP trainee (GPVTS)	55 (9.0%)
	Specialty trainee/Registrar (ST3+)	83 (13.6%)
	Other	84 (13.7%)

Distribution of medical students (*N* = 1757) across undergraduate and graduate-entry year groups, and resident doctors (*N* = 612) across UK postgraduate training stages including FY1–FY2, CT1–CT3, IMT, GPVTS, ST3+, and other pathways. Values are presented as *n* (%).

**Table 2:** Gender and ethnicity distribution of medical students and resident doctors.

		Resident doctors (*n* = 612)	Medical students (*n* = 1757)
Gender	Female	323 (52.8%)	1059 (60.3%)
	Male	271 (44.3%)	636 (36.2%)
	Non-binary/Third gender	4 (0.7%)	33 (1.9%)
	Prefer not to say	14 (2.3%)	29 (1.7%)
Ethnicity	Asian or Asian British	296 (48.4%)	727 (41.4%)
	Black or Black British	41 (6.7%)	214 (12.2%)
	Mixed/Multiple ethnic groups	39 (6.4%)	162 (9.2%)
	White	176 (28.8%)	546 (31.1%)
	Other	31 (5.1%)	57 (3.2%)
	Prefer not to say	29 (4.7%)	51 (2.9%)

Self-reported gender and ethnicity of medical students (*N* = 1757) and resident doctors (*N* = 612). Data are presented as counts and percentages for each demographic category.

### Undergraduate and postgraduate educational and clinical exposure to aesthetics

Among medical students, only 10.9% (*n* = 191) reported receiving formal teaching within their medical school curriculum, and 13.2% (*n* = 232) reported exposure outside of medical school. Among resident doctors, 10.5% (*n* = 64) reported receiving undergraduate aesthetic teaching, while postgraduate exposure remained limited, with 11.9% (*n* = 73) undertaking some form of aesthetic training (see Supplementary Figure 2 for a full breakdown of postgraduate training). Cross-tabulations of undergraduate and postgraduate teaching are presented in Tables 3a and b.

**Table 3:** (a-b) Cross-tabulation of aesthetic teaching exposure among resident doctors and medical students.

		Postgraduate Training		
		Yes	No	Total
Formal teaching in med school	Yes	14	50	64
	No	59	489	548
	Total	73	539	612
		Outside of Med School		
		Yes	No	Total

Formal teaching in med school	Yes	62	129	191
	No	170	1396	1566
	Total	232	1525	1757

These tables summarize patterns of aesthetic education across training stages. Table 3a presents the relationship between resident doctors’ (*N* = 612) exposure to formal undergraduate aesthetic teaching and their receipt of postgraduate aesthetic training. Table 3b shows the relationship between medical students’ (*N* = 1757) formal aesthetic teaching within the medical school curriculum and additional teaching obtained outside medical school. Cell values represent the number of participants reporting each combination of teaching exposure, with row and column totals indicating overall uptake at each stage.

**Figure 1:**
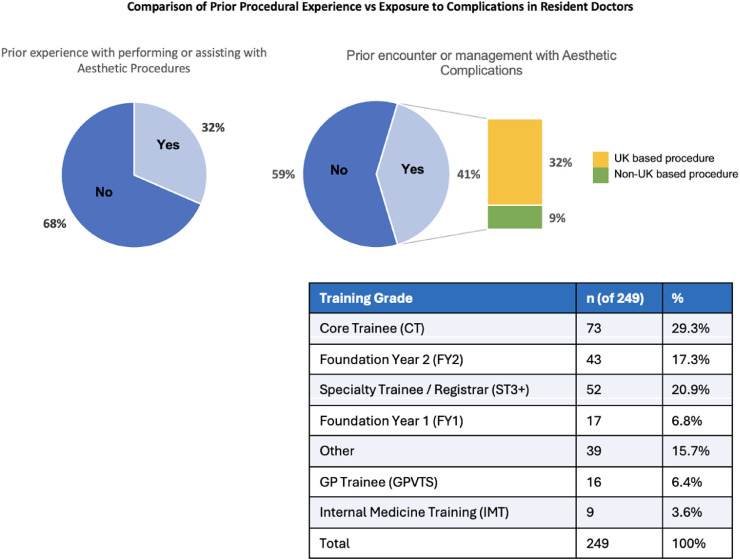
Prior procedural experience and geographic origin of aesthetic complications among resident doctors. Proportion of resident doctors (*N* = 612) reporting prior experience assisting with or performing aesthetic procedures, alongside the number of resident doctors who had managed aesthetic complications (*n* = 249). Complications are additionally categorized by whether the original procedure was performed within the UK or outside the UK and also the training grade of the doctors who responded that they had managed a complication.

Procedural experience among resident doctors was limited, with only 31.5% (*n* = 193) having assisted with or performed aesthetic procedures. Management of aesthetic complications was more common: 40.7% (*n* = 249) of resident doctors had managed at least one complication. Of the 249 resident doctors, 158 were doctors working outside higher surgical training, including junior trainees such as FY and IMT doctors. Of the complications encountered by resident doctors, the majority (*n* = 195; 78.3%) arose from procedures performed outside the UK (see Figure 1).

### Knowledge of procedures

Among students, surgical knowledge ranged from 1.72 ± 1.02 (genioplasty) to 2.57 ± 1.25 (rhinoplasty), while non-surgical knowledge ranged from 2.37 ± 1.32 (chemical peels) to 2.82 ± 1.32 (botulinum toxin). Doctors’ surgical knowledge ranged from 1.73 ± 1.12 (genioplasty) to 2.65 ± 1.39 (breast augmentation), while non-surgical knowledge ranged from 2.32 ± 1.33 (chemical peels) to 2.99 ± 1.37 (botulinum toxin). Full breakdown of knowledge scores can be found in Supplementary Table 2.

### Perceptions of aesthetic practice

A majority of both medical students (74.2%, *n* = 1304) and resident doctors (69.1%, *n* = 423) recognized the positive impact of aesthetic surgery on patient quality of life and acknowledged the field’s importance to contemporary medical practice (doctors 63.2%, *n* = 387; students 64.8%, *n* = 1139).

Participants also identified challenges in professional perception, presenting a barrier to workforce development: fewer than 40% in either group (resident doctors 33.3%, *n* = 204; students 38.9%, *n* = 700) felt the specialty is respected within the wider medical community, while approximately two-thirds of both groups (resident doctors 66.3%, *n* = 406; students 68.8%, *n* = 1208) reported awareness of stigma surrounding the pursuit of an aesthetics career (see Table 4 for a full breakdown of attitudes across all statements).

**Table 4:** Participant perceptions of aesthetic surgery and the aesthetic industry.

Statement	Group	Strongly disagree (% (*n*))	Disagree (% (*n*))	Neutral (% (*n*))	Agree (% (*n*))	Strongly agree (% (*n*))
Complications in aesthetic procedures are more distressing to patients than complications in non-aesthetic procedures.	Resident doctor	5.2% (32)	11.4% (70)	23.4% (143)	39.2% (240)	20.8% (127)
	Medical student	4.3% (75)	10.8% (189)	40.4% (709)	33.2% (584)	11.4% (200)
Patients undergoing aesthetic procedures face greater societal judgment when complications occur.	Resident doctor	1.5% (9)	4.1% (25)	12.4% (76)	46.1% (282)	35.9% (220)
	Medical student	1.6% (28)	5.7% (100)	21.8% (383)	39.5% (694)	31.4% (552)
Psychological impacts of complications are more severe in aesthetic procedures than in non-aesthetic procedures.	Resident doctor	3.6% (22)	10.5% (64)	25.5% (156)	38.2% (234)	22.2% (136)
	Medical student	3.2% (56)	11% (193)	37.7% (662)	34.2% (601)	13.9% (245)
Aesthetic surgery significantly improves patient quality of life.	Resident doctor	1.3% (8)	7.7% (47)	21.9% (134)	48.5% (297)	20.6% (126)
	Medical student	1.1% (20)	3.5% (61)	21.2% (372)	51.9% (912)	22.3% (392)
Aesthetic surgery is an essential part of plastic and reconstructive surgery.	Resident doctor	1.6% (10)	8.3% (51)	17.3% (106)	38.6% (236)	34.2% (209)
	Medical student	0.9% (16)	3.8% (66)	24.2% (425)	44.6% (784)	26.5% (466)
Aesthetic surgery is an important field in medicine.	Resident doctor	3.9% (24)	13.6% (83)	19.3% (118)	39.1% (239)	24.2% (148)
	Medical student	1.6% (28)	6.1% (108)	27.4% (482)	43.9% (771)	20.9% (368)
Aesthetics is a respected field within the medical community.	Resident doctor	11.1% (68)	30.7% (188)	24.8% (152)	25.7% (157)	7.7% (47)
	Medical student	3.2% (56)	21.6% (379)	35.4% (622)	29.7% (521)	10.2% (179)
Aesthetics is a respected field amongst members of the wider public.	Resident doctor	6.7% (41)	22.2% (136)	27.3% (167)	34.6% (212)	9.2% (56)
	Medical student	3.3% (58)	17.6% (309)	37.1% (652)	31.4% (551)	10.6% (187)
There is stigma around pursuing a career in aesthetic surgery.	Resident doctor	3.3% (20)	11.1% (68)	19.3% (118)	43.1% (264)	23.2% (142)
	Medical student	2.2% (39)	6.7% (117)	22.5% (396)	39.3% (691)	29.3% (514)
The high financial cost of aesthetic surgery training influences its accessibility for trainees.	Resident doctor	1.5% (9)	4.4% (27)	20.8% (127)	43.1% (264)	30.2% (185)
	Medical student	1.7% (29)	5.5% (97)	25.8% (453)	36.3% (637)	30.8% (541)
Aesthetic surgery is a profit-driven industry.	Resident doctor	2.3% (14)	5.4% (33)	15.4% (94)	42.6% (261)	34.3% (210)
	Medical student	1.9% (34)	6.5% (114)	26.2% (460)	42.4% (745)	23% (404)

Responses from resident doctors (*N* = 612) and medical students (*N* = 1757) to statements regarding attitudes toward aesthetic practice, patient outcomes, stigma, commercialization, and professional respect. Results are presented as percentages and counts across a five-point Likert scale ranging from “strongly disagree” to “strongly agree.”

**Figure 2:**
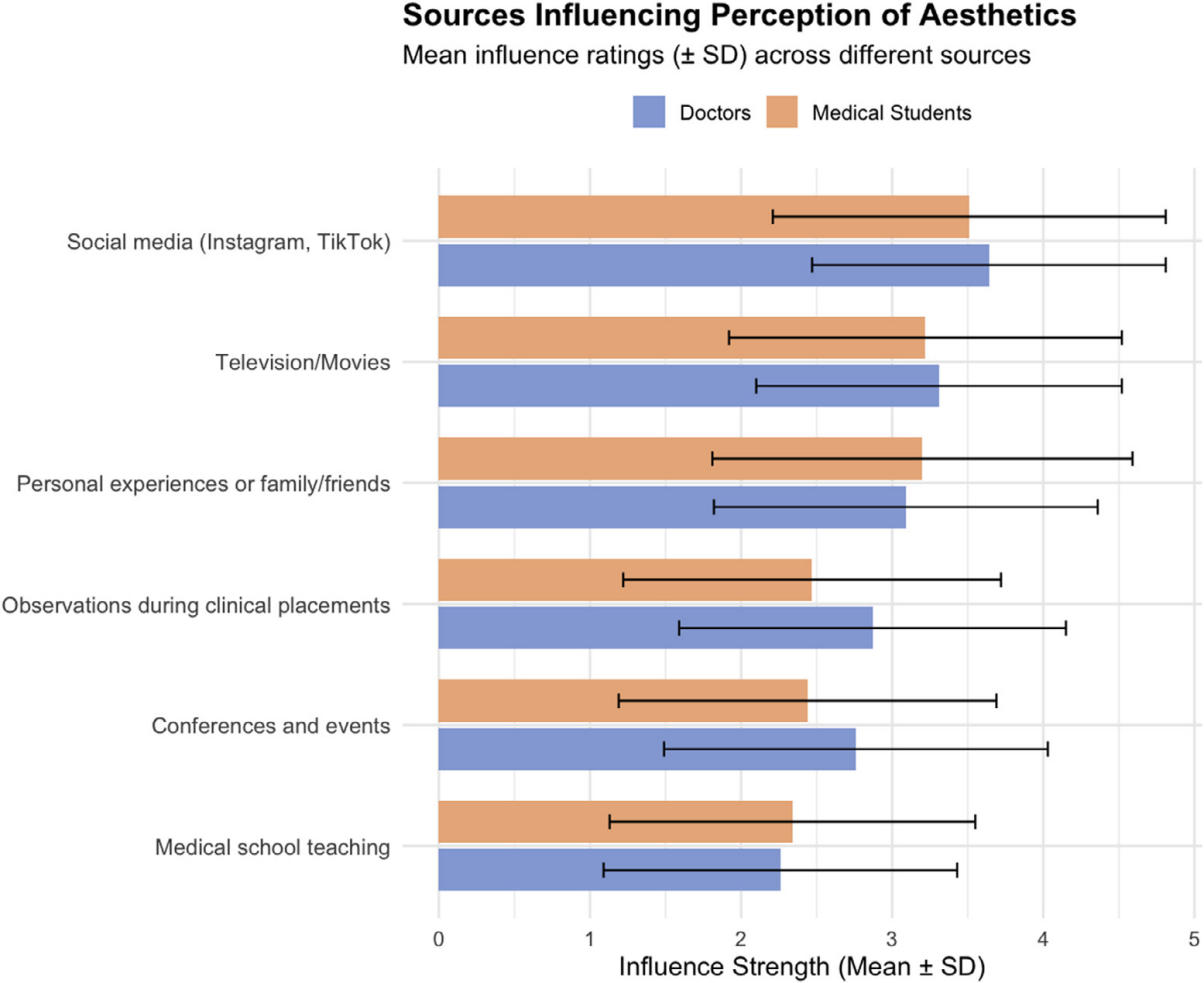
Influence of information sources on perceptions of aesthetic practice. Mean influence scores (±SD) for various information sources shaping participant perceptions of the aesthetic industry.

Across both cohorts, perceptions of aesthetic surgery were shaped predominantly by external sources rather than formal educational platforms. Social media was the single most influential source for both groups (doctors 3.64 ± 1.17; students 3.51 ± 1.30), with formal medical training rated the least influential (resident doctors 2.26 ± 1.17; students 2.34 ± 1.21) (see Figure 2).

### Barriers and motivation to pursuing a career in aesthetics

**Figure 3:**
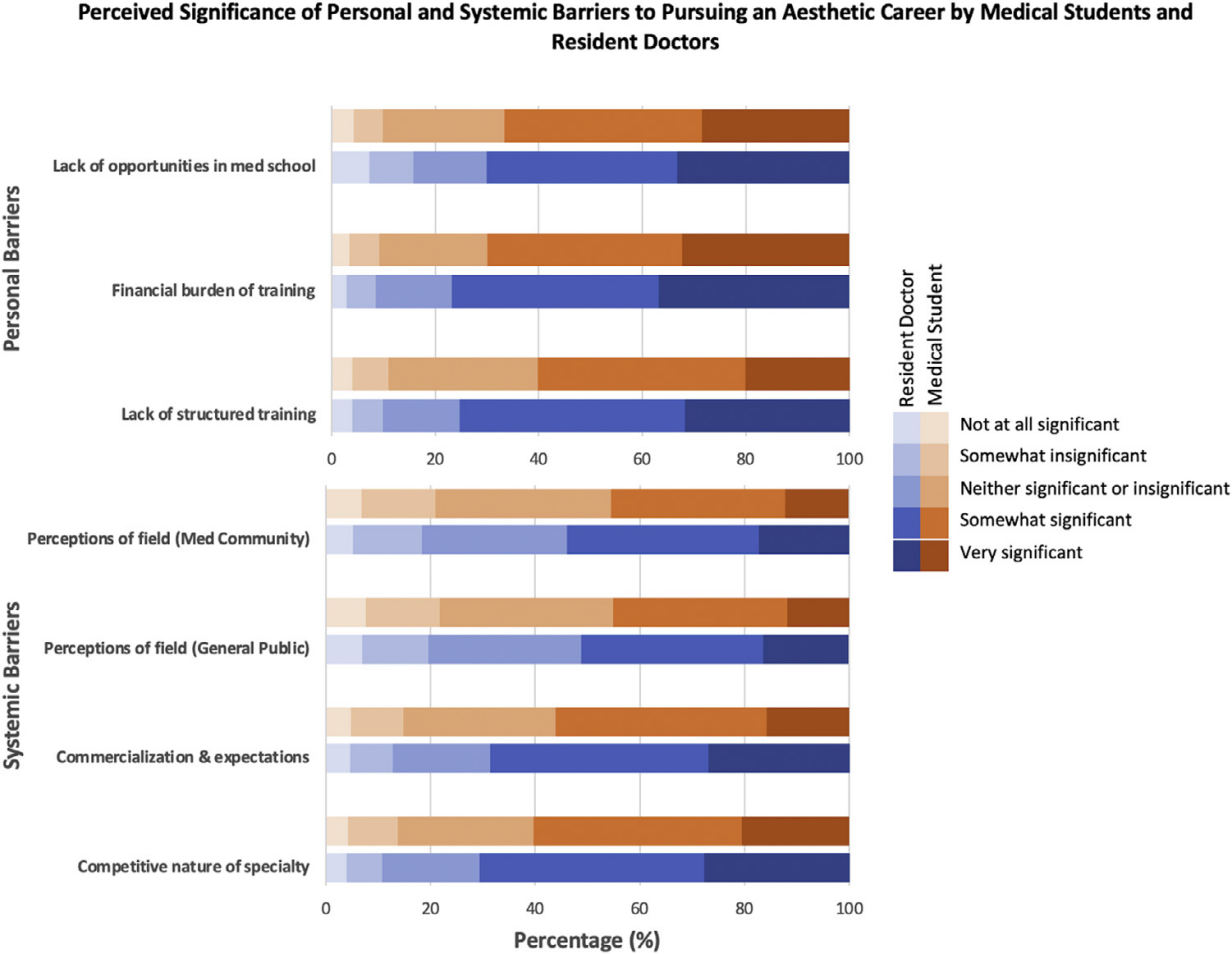
Perceived barriers to pursuing a career in aesthetic practice. Proportion of medical students (*N* = 1757) and resident doctors (*N* = 612) identifying key personal and systemic barriers to entering aesthetic practice.

Financial burden was the most frequently cited personal barrier (doctors 76.8%, *n* = 470; students 70.0%, *n* = 1230), followed by lack of structured training (doctors 75.4%, *n* = 461; students 60.3%, *n* = 1059). The competitive nature of the specialty was viewed as the most significant systemic barrier (doctors 70.8%, *n* = 420; students 60.3%, *n* = 1060), followed by concerns about commercialization (doctors 68.7%, *n* = 433; students 56.1%, *n* = 985) (see Figure 3).

Interest in pursuing a career in aesthetics was expressed by 36.5% of students (*n* = 642) and 43.0% of doctors (*n* = 263). Detailed motivating factor distributions for those expressing career interest are provided in Supplementary Figure 3.

### Ethical concerns regarding the aesthetic industry

**Figure 4:**
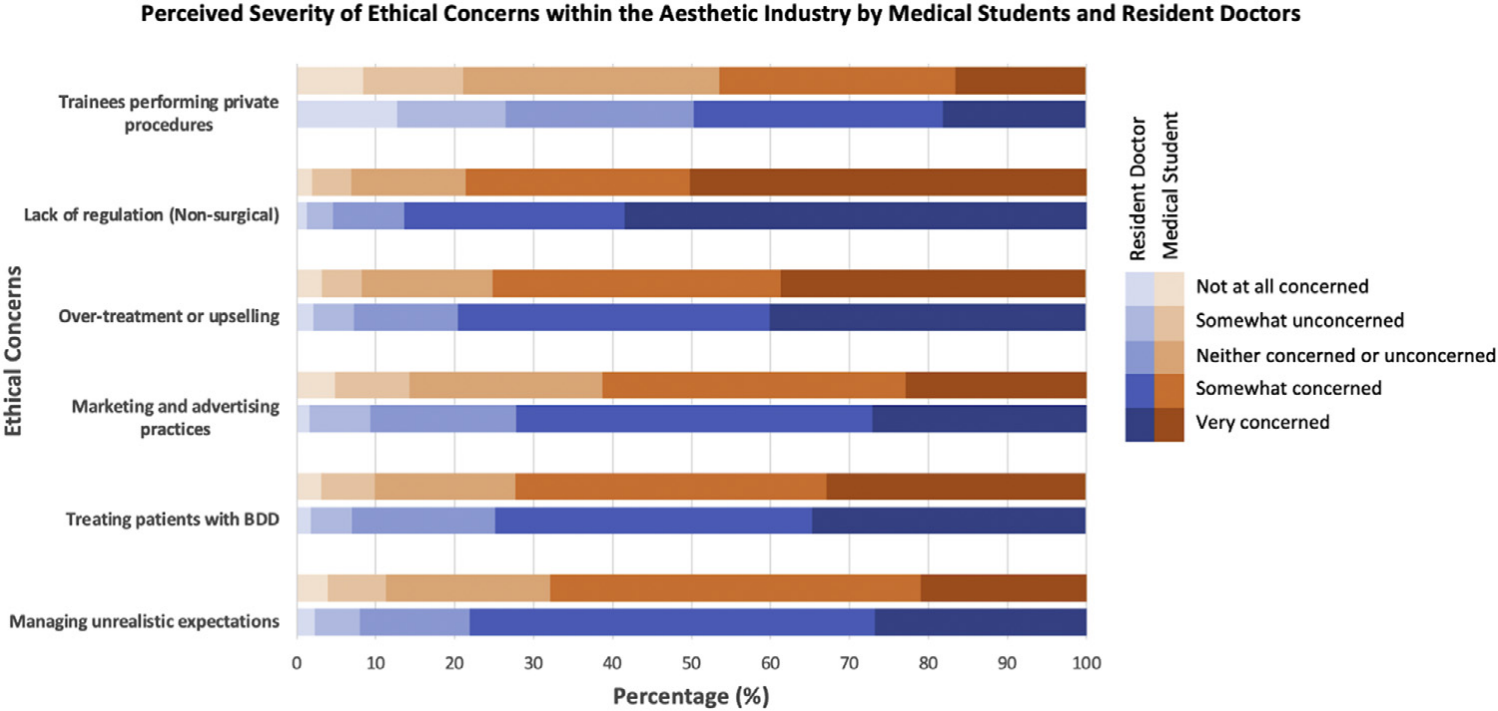
Ethical concerns identified by participants regarding the aesthetics industry. Proportion of medical students and resident doctors rating each ethical concern on a five-point Likert scale.

Resident doctors consistently reported greater ethical concerns regarding the aesthetics industry than students. Over-treatment was flagged as a significant concern regarding patient welfare (doctors 79.5%, *n* = 487; students 75.1%, *n* = 1319), followed by treating patients with body dysmorphic disorder (BDD) (doctors 74.8%, *n* = 458; students 72.2%, *n* = 1270) (see Figure 4).

**Figure 5:**
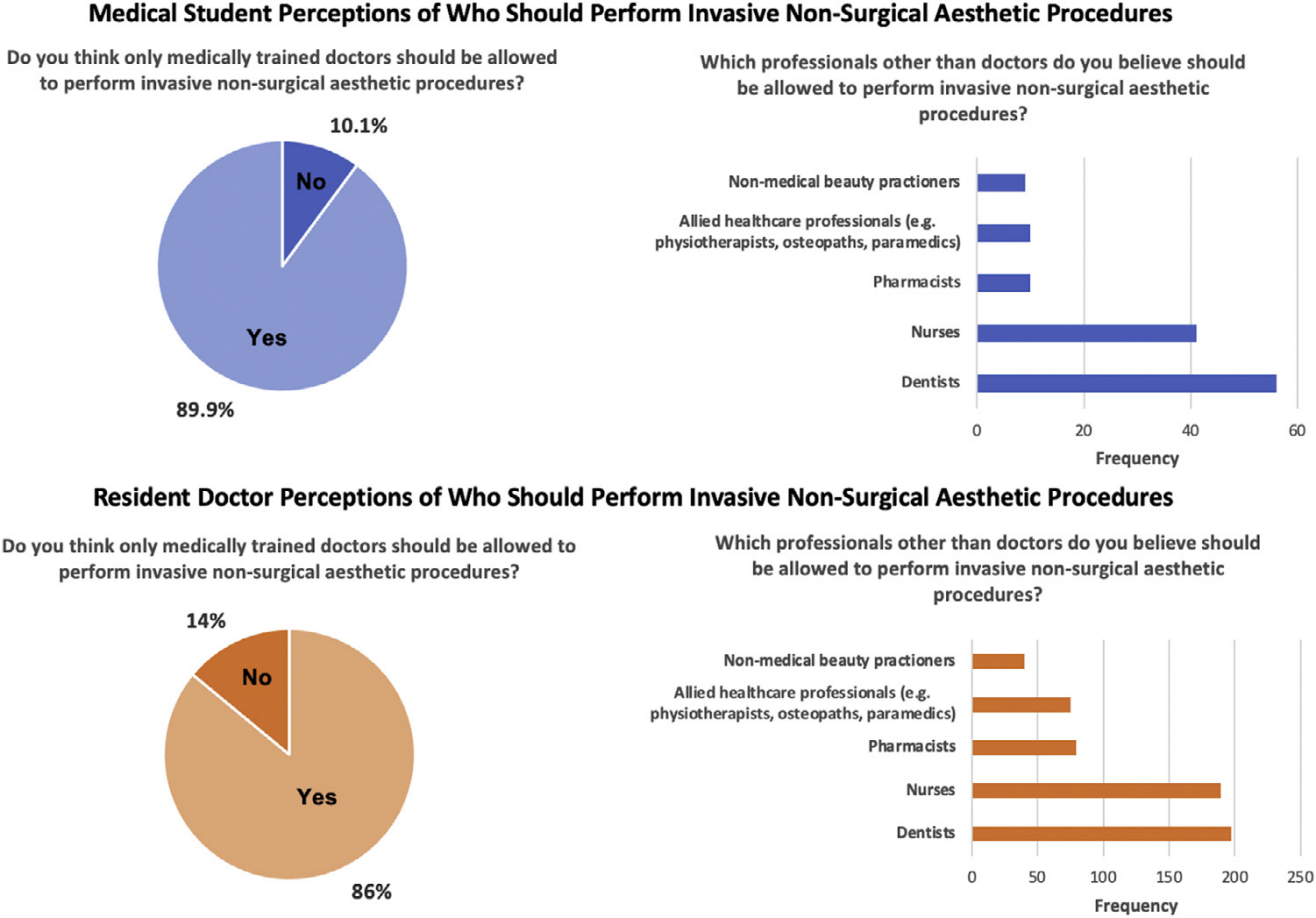
Participant views on who, other than medically trained doctors, should be permitted to perform invasive non-surgical aesthetic procedures. Responses from medical students (*N* = 1757) and resident doctors (*N* = 612) indicating whether invasive non-surgical aesthetic procedures should be performed only by doctors. For participants who did not support doctor-only provision (students *n* = 246; residents *n* = 62), the figure displays their selected alternative professional groups considered appropriate to perform such procedures.

Lack of regulation in the non-surgical sector emerged as the most prominent concern, rated as significant by 86.4% (*n* = 550) of resident doctors and 78.6% (*n* = 1382) of students. There was strong support for restricting invasive non-surgical aesthetic procedures to medically trained doctors (doctors 88.2%, *n* = 540; students 86.0%, *n* = 1511). The small proportion of respondents in each group who felt that other professionals (e.g., dentists, nurses, allied health practitioners) could undertake these procedures is summarized in Figure 5.

### Views on training and curricula

Both groups expressed low agreement that aesthetic medicine and surgery are well integrated into medical school curricula (medical students: 1.88 ± 1.12; residents: 1.78 ± 1.11) (see Supplementary Figure 4).

**Figure 6:**
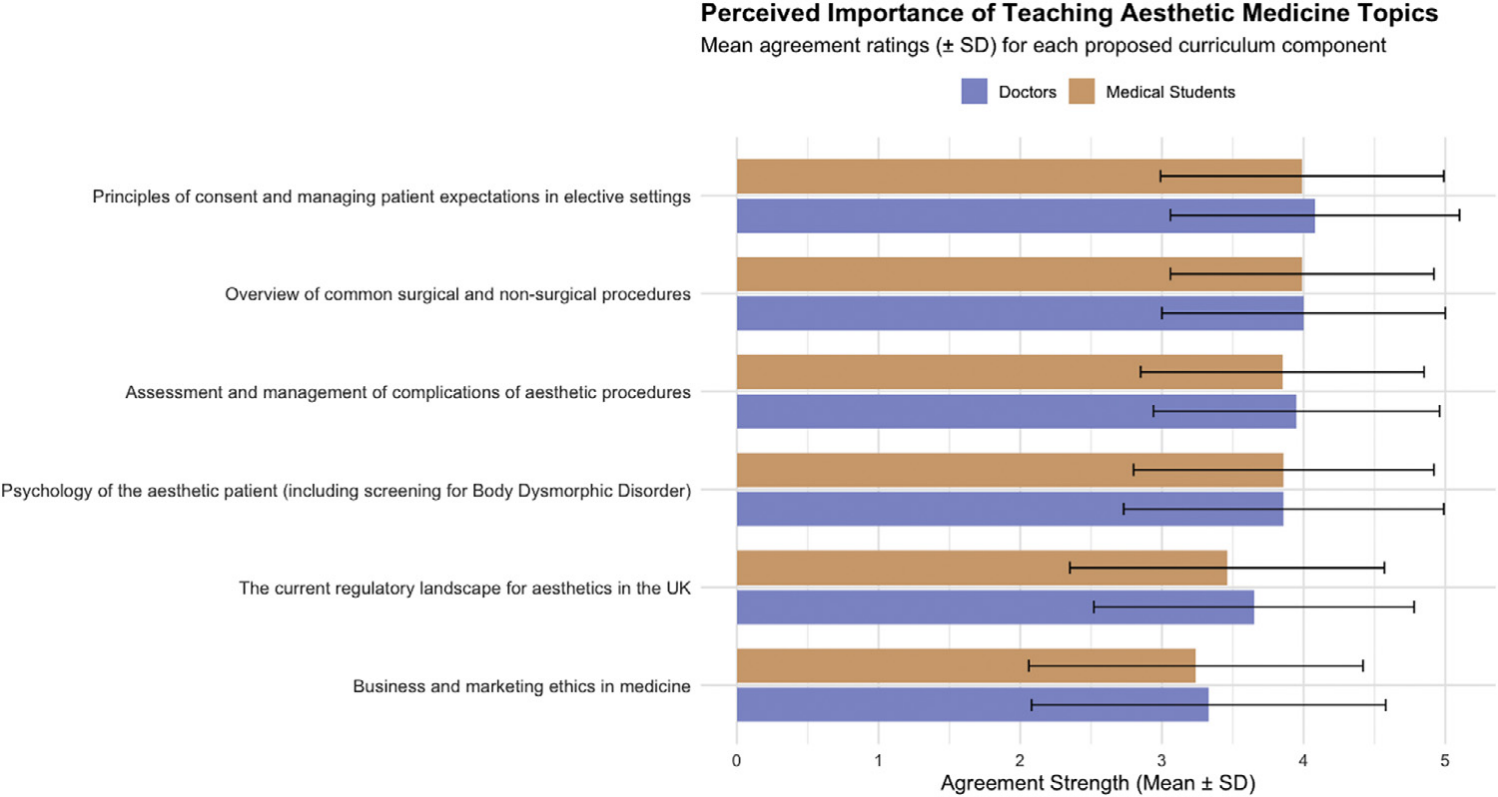
Preferred topics for inclusion in undergraduate aesthetic education. Mean participant ratings (±SD) of proposed aesthetic curriculum topics for inclusion within undergraduate medical training.

Participants prioritized principles of consent and managing patient expectations as the most desirable topic for incorporation within undergraduate curriculum (doctors, 4.08 ± 1.02; students, 3.99 ± 1.0), followed by an overview of common aesthetic procedures (doctors, 4.00 ± 1.00; students, 3.99 ± 0.93). Psychology of the aesthetic patient was also rated highly (doctors, 3.86 ± 1.13; students, 3.86 ± 1.06). Full rankings are shown in Figure 6.

## Discussion

### Principal findings

10.8% of participants reported receiving formal aesthetic education at medical school, while 11.9% of resident doctors reported postgraduate exposure. This was reflected in the self-reported knowledge scores in both groups, ranging from 1.72 to 2.65/5 for surgical procedures and 2.32–2.99/5 for non-surgical procedures, consistent with the low rates of curriculum integration reported. These scores were notably lower for surgical procedures than non-surgical ones, suggesting that non-surgical content may be more accessible or informally encountered, perhaps through media exposure, though formal teaching remains minimal across both domains.

Despite these low levels of familiarity, 40.7% of resident doctors had already encountered and managed at least one aesthetic complication, with 63.5% of these clinicians being junior residents outside of higher surgical training. It is important to note, however, that the study did not capture the specific clinical context in which these complications were managed, including whether doctors had previously rotated through surgical posts, which limits conclusions about the breadth of cross-specialty exposure. Furthermore, no data were collected on the specific nature of complications encountered, which constrains inference about the complexity of management required. These findings nonetheless suggest that awareness of aesthetic complications and appropriate escalation pathways may be relevant beyond specialist surgical training alone.

The high proportion (78.3%) of managed complications arising from procedures performed abroad is consistent with previously reported trends in cosmetic tourism and its burden on NHS resources.^16^^,^^17^ This reinforces the importance of preparing clinicians to recognize and manage complications from diverse procedural and regulatory contexts.

Engagement with the specialty was substantial, with 38.2% of participants expressing interest in pursuing a career in aesthetics. However, participants identified several systemic challenges to training engagement, most commonly financial burden (doctors 76.8%; students 70.0%) and lack of structured training pathways (doctors 75.4%; students 60.3%). In parallel, there was strong professional consensus in favor of enhanced regulation, particularly within the non-surgical sector (doctors 86.4%; students 78.6%). The majority of participants were in favor of restricting these procedures to medical professionals (doctors 88.2%; students 86.0%).

### Comparison with existing literature

Previous studies examining student and trainee perspectives on aesthetic education report findings that largely align with those of the SCULPT study, suggesting that many challenges have persisted over time. At undergraduate level, medical students consistently report minimal formal exposure to both surgical and non-surgical aesthetics, accompanied by low knowledge and confidence, despite strong interest in the field.^18^^,^^26^ These findings mirror those of this study, which identified limited undergraduate exposure and modest self-reported knowledge across aesthetic domains.

Within postgraduate training, multiple studies highlight ongoing structural limitations and variability in access to aesthetic learning. Plastic surgery trainees report limited exposure, particularly in facial aesthetics, restricted access to formal training opportunities, and reliance on fellowships or private-sector experience to achieve relevant competencies.^27^, ^28^, ^29^ There is also a call among trainees for formal integration of aesthetic training into structured programmes.^28^ Beyond plastic surgery, there was substantial variation in aesthetic content across surgical curricula, with OMFS and ENT trainees also reporting limited exposure and similar concerns regarding accessibility of training resources.^30^ Collectively, these findings closely reflect our participants’ reports of financial and structural barriers to training, as well as restricted access to formal aesthetic education.

### Future directions

Future work should explore the development and evaluation of structured, safety-focused aesthetic education, with curriculum content differentiated by the specific needs and scope of practice of different learner groups. Given that a substantial proportion of resident doctors in this study reported encountering aesthetic complications, future research should investigate the specific clinical contexts in which this occurs, to better delineate which competencies are most relevant to generalist versus specialist training. For early curriculum integration, core competencies of potential relevance to a broad clinical audience include recognition of aesthetic complications, appropriate escalation pathways, principles of consent, patient expectation management, and psychological assessment,^31^^,^^32^ with particular attention to safety-critical content such as applied anatomy, recognition of vascular occlusion and infection, and appropriate escalation pathways, including screening for BDD.^32^^,^^33^

The influence of social media on perceptions of aesthetics was a recurring theme, aligning with participants’ concerns about marketing practices and overtreatment or upselling of procedures. Training should enable future and current clinicians to critically appraise online content, counsel patients with idealized expectations, and communicate realistic outcomes and risks.

At postgraduate level, future research should explore equitable training pathways addressing the financial and structural barriers identified by participants.^31^ Collaboration between NHS services and accredited private providers may offer a potential avenue for supervised clinical exposure,^31^^,^^34^ as demonstrated during the COVID-19 pandemic. Expanding mentorship schemes to early-stage trainees may support training quality, patient safety, and governance standards, alongside accredited training centers and competency-based progression models.^31^^,^^35^

The high proportion of complications arising from cosmetic tourism underscores its ongoing impact on NHS workload and resource utilization. Longitudinal and multicenter studies could inform targeted educational resources, clinical guidance, and referral pathways.^31^^,^^34^

Future policy-oriented research should build on professional consensus regarding the need for better regulation, particularly within the non-surgical aesthetic sector. Evaluation of national accreditation frameworks, practitioner registration, credentialing systems, and CPD-based revalidation will be essential to ensure consistent educational quality, appropriate scope of practice, and risk-based regulation linked to procedural invasiveness.^31^^,^^35^, ^36^, ^37^, ^38^

### Strengths and implications

The SCULPT study provides the first national characterization of exposure to, and perceptions of, aesthetic medicine and surgery across UK undergraduate and postgraduate training. Unlike prior studies limited to a single specialty or institutions, this study reflects the strong multidisciplinary nature and growing clinical relevance of aesthetic practices with the inclusion of both medical students and resident doctors across a broad range of grades and specialties. While existing literature has primarily focused on educational exposure, this study further evaluates knowledge, attitudes, career interest, perceived barriers, and views on regulation. Through a more holistic perspective, it therefore provides a clear blueprint for action to inform curriculum development, accessibility to training pathways in aesthetic practice, and policy initiatives aligned with professional standards and enhancing patient safety.

### Limitations

As a cross-sectional, self-reported survey, this study reflects participants’ views at a single point in time and may be influenced by recall or social desirability bias. A formal response rate could not be calculated given the convenience sampling approach via social media and professional networks, meaning the total number of individuals reached was not ascertained. Consequently, selection bias remains a significant limitation: participants with an interest in aesthetics may have been more likely to respond, and findings may not be generalizable to the broader UK medical student and resident doctor population. We are also unable to report on the number of survey clicks. Although the survey was disseminated nationally across 44 medical schools and multiple postgraduate training regions, some institutions were underrepresented, and no international comparisons were included. Use of self-reported data means that actual knowledge or clinical competence cannot be objectively assessed.

The survey did not systematically differentiate between surgical and non-surgical aesthetic procedures in all sections, which limits the extent to which specialty-specific educational needs can be delineated from these data. Data on the specific clinical context in which resident doctors managed aesthetic complications (e.g., prior surgical rotations versus current post, the clinical setting in which it was encountered) were not collected, limiting causal inference about the cross-specialty burden of aesthetic complications.

## Conclusion

As the aesthetic healthcare sector continues to expand, this study offers timely evidence to support the evolution of education, training, and regulation. By aligning educational provision and professional standards with the realities of contemporary aesthetic practice, there is an opportunity to better prepare clinicians, support patients navigating aesthetic decisions, and strengthen patient safety within both NHS and non-NHS settings. Addressing these priorities is essential to the safe, ethical, and professional development of aesthetics within modern medical practice.

## List of abbreviations

**Table:** 

SCULPT	Student and Doctor Comprehension, Uptake of Learning, and Perception of Aesthetic Surgery and Training
GMC	General Medical Council
RCS	Royal College of Surgeons
BAAPS	British Association of Aesthetic Plastic Surgeons
NHS	National Health Service
UKPRC	United Kingdom Plastics Research Collaborative
PBAS	Professional Behaviors in Aesthetic Surgery
PRISMA-ScR	Preferred Reporting Items for Systematic Reviews and Meta-Analyses extension for Scoping Reviews
STROBE	Strengthening the Reporting of Observational Studies in Epidemiology
GDPR	General Data Protection Regulation
ISAPS	International Society of Aesthetic Plastic Surgery
OMFS	Oral and Maxillofacial Surgery
CPD	Continuing Professional Development
ENT	Ear, Nose & Throat
BDD	Body Dysmorphic Disorder

## Availability of data and materials

The datasets generated and analyzed during the current study are available from the corresponding author (UR) upon reasonable request. The scoping review and full survey instrument are included as Supplementary Appendix A and B respectively. Other supplementary results are also provided on medical student perceptions of accessibility of aesthetics (Supplementary Figure 5), perceived technical demand of surgical and non-surgical aesthetic procedures (Supplementary Figure 6), and preferred specialty pathways for pursuing aesthetic practice (Supplementary Figure 7).

## Ethical approval and consent to participate

Ethical approval was granted by the Imperial College Education Ethics Review Process (EERP2425-128, 28/07/2025). Participation was voluntary, with electronic informed consent obtained. The information sheet outlined confidentiality and right to withdraw before submission. No identifiable data were collected and responses were accessible only to the lead researchers. Data storage complied with UK General Data Protection Regulation (GDPR) and the Declaration of Helsinki.

## Funding

This research did not receive any specific grant from funding agencies in the public, commercial, or not-for-profit sectors.

### Declaration of generative AI and AI-assisted technologies in the writing process

During the preparation of this manuscript, the authors used ChatGPT (OpenAI) to assist with improving clarity and readability of the text. The authors critically reviewed and edited all content generated using this tool and take full responsibility for the integrity, accuracy, and originality of the work.

## Data sharing

The data that support the findings of this study are available on request from the corresponding author (UR).

## Declaration of competing interest

The authors declare they have no competing interests.

## Appendix Supplementary materials

Image, application 1

Image, application 2

Image, application 3

Image, application 4

Image, application 5

Image, application 6

Image, application 7

Image, application 8

Image, application 9

Image, application 10

Image, application 11
