# Supplementary material for: SCULPT: Medical student and resident doctor comprehension, uptake of learning and perception of aesthetic surgery and training
Source: JPRAS Open. 2026 Apr 4;50:10–25. doi: 10.1016/j.jpra.2026.03.043 (PMC13127476; doi:10.1016/j.jpra.2026.03.043)
Supplement: Supplementary file 1 [file mmc1.docx]

Appendix A: Scoping Review Protocol and Results

# **Protocol and Search Strategy**

This scoping review was conducted in accordance with the PRISMA-ScR (Preferred Reporting Items for Systematic Reviews and Meta-Analyses extension for Scoping Reviews) guidelines^1^. The aim was to map the existing literature concerning aesthetic surgery and medicine training for UK medical students and doctors in training, identify key themes, and highlight gaps in research to inform the development of the SCULPT survey tool. The protocol for this study was registered on PROSPERO (Registration Number: CRD42025120728).

## **Search Strategy**

Three electronic databases, PubMed, Embase, and Scopus, were systematically searched with no restriction on publication dates. The search was executed on [Insert Date, e.g., 15th November 2025]. The following search terms and Boolean operators were used:

("UK" OR "United Kingdom") AND ("aesthetic surgery" OR "aesthetic medicine" OR "aesthetics" OR "cosmetic surgery" OR "cosmetic medicine" OR "cosmetic procedures" OR "non-surgical aesthetics") AND ("medical students" OR "medical school" OR "undergraduate medical education" OR "resident doctors" OR "junior doctors" OR "foundation doctors" OR "doctors in training" OR "surgical trainees" OR "surgeons").

# **Eligibility Criteria**

**Appendix A Table 1. Inclusion and exclusion criteria for the scoping review**

Summary of eligibility criteria applied to identify relevant studies examining aesthetic surgery and medicine education, training, perceptions, and regulation among UK medical students and resident doctors.

| **Inclusion Criteria** | **Exclusion Criteria** |
| --- | --- |
| - Original research study designs (e.g., quantitative, qualitative, mixed-methods studies). - Studies published in the English language. - Studies focusing on UK-based populations, including undergraduate medical students and resident doctors. - Studies discussing any aspect of aesthetic surgery or aesthetic medicine education, training, perceptions, or regulation. - Studies exploring curriculum development, ethical considerations, or challenges/barriers related to aesthetics training for the target population. - Studies exploring cosmetic tourism and media use within the aesthetic industry | - Reviews and conference abstracts. - Studies published in non-English languages. - Studies focused on non-UK populations or training systems. - Articles focusing exclusively on perceptions of non-medical practitioners (e.g., dental, nursing, or beauty therapy aesthetics) without relevance to medical or surgical trainees. - Studies lacking specific data relevant to aesthetic surgery training or education for doctors. |

# **Study Selection and Data Extraction**

## **Selection Process**

Two independent reviewers (AS, RS) screened the titles, abstracts, and full texts of the articles using the Covidence platform. Discrepancies were resolved through discussion with a senior reviewer. A record of inclusion/exclusion decisions was maintained in Microsoft Excel. In cases of missing or unclear data, corresponding authors were contacted for clarification.

## **PRISMA Flow Diagram**

**Appendix A Figure 1. PRISMA flow diagram for study selection in the scoping review**

Process of identification, screening, eligibility assessment, and inclusion of studies in accordance with PRISMA-ScR guidelines.


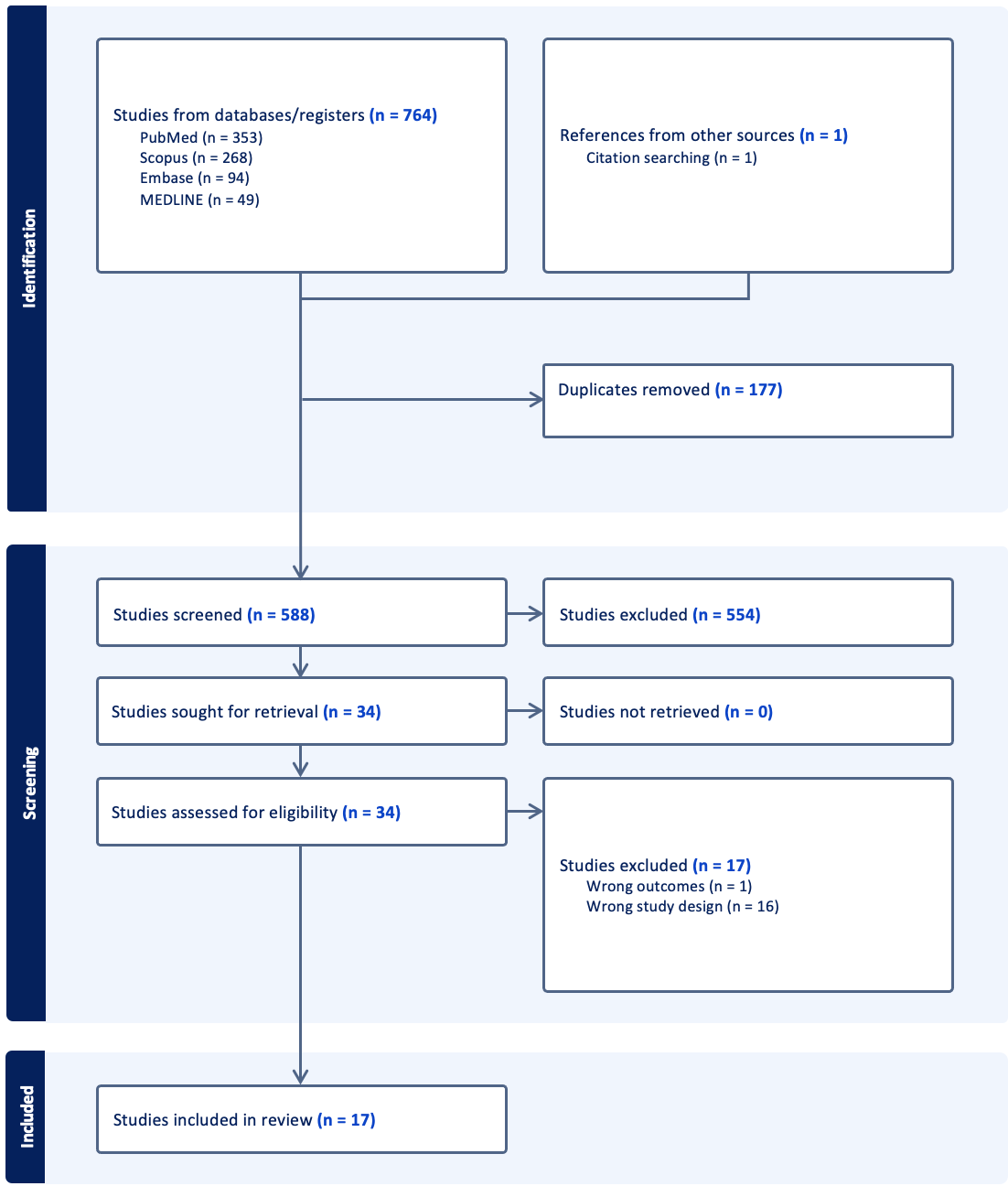


## **Data Synthesis and Reporting**

Data extraction was conducted independently by two reviewers using a standardised Excel spreadsheet. Key study characteristics such as author, publication year, and study design were recorded, alongside study population and sample size. Critical appraisal of individual evidence sources was not performed, as the primary objective was to map key themes for survey development rather than to assess the methodological quality of existing literature .

Extracted content was systematically mapped to pre‑defined themes encompassing training and curriculum design, perceptions and attitudes toward aesthetics, regulatory considerations, ethical practice, media influences, cosmetic tourism, barriers to training and curriculum implementation. Principal findings, conclusions, and recommendations for policy, practice, and further research were summarised to shape the domains of the survey instrument.The domains are depicted in Appendix Figure 2.

**Appendix A Figure 2. Domains of the SCULPT survey instrument derived from the scoping review**

Conceptual mapping of key themes identified in the scoping review to the final domains incorporated into the SCULPT survey instrument.

#
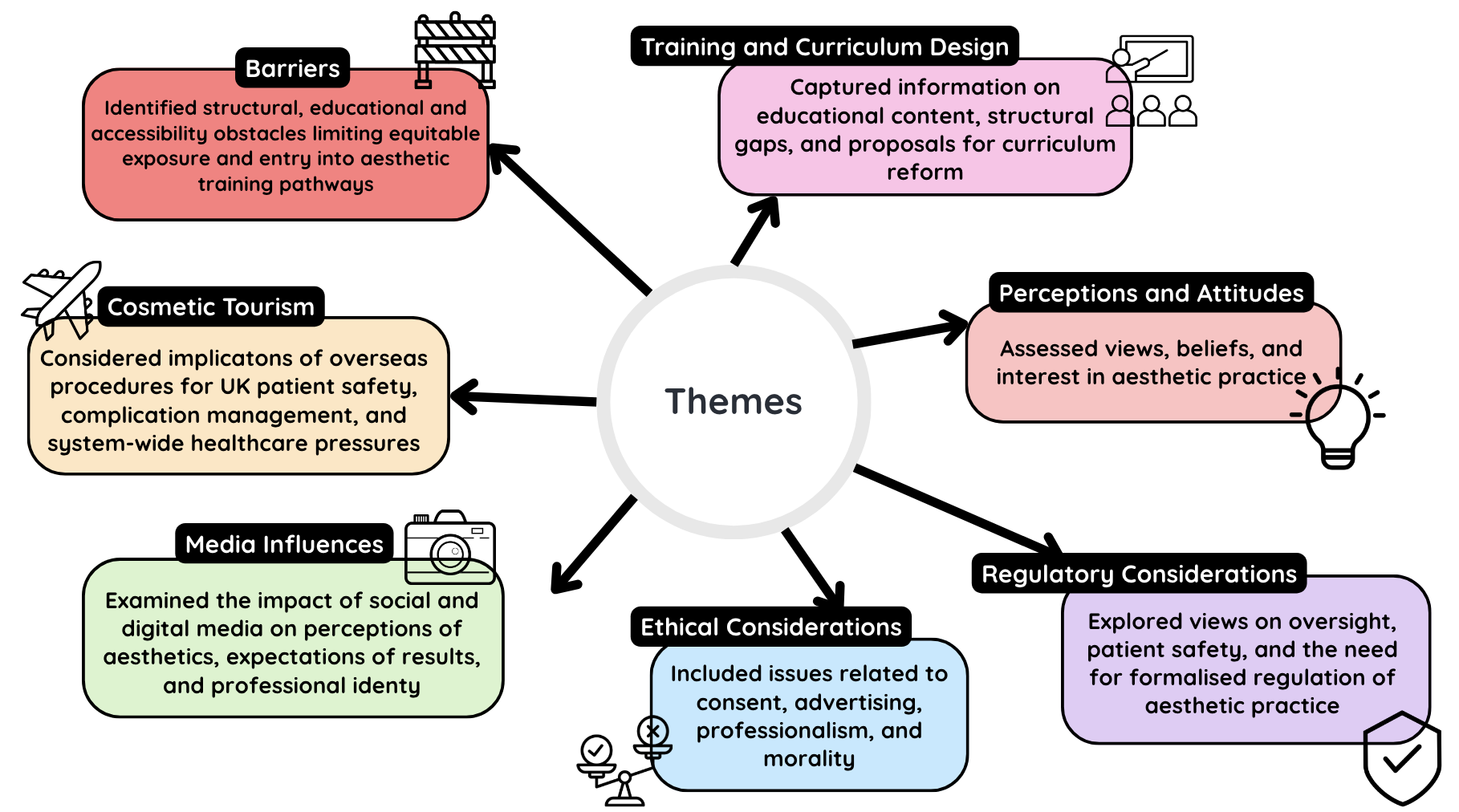


# **Results**

The search criteria identified 588 studies, 17 of which met the full-text inclusion criteria. Six dominant themes informed survey development: student and trainee perceptions and attitudes toward aesthetics (n=6), training and curriculum design (n=7), ethical practice (n=4), regulatory considerations (n=4), media influence (n=5), cosmetic tourism (n=2), and barriers to training (n=2).

**Appendix A Table 2. Summary of included studies and key domains informing SCULPT survey development**

Characteristics and extracted data from included studies, including study design, sample size, key themes, key findings, and relevance to SCULPT survey domain development

| **Study** | **Study Design** | **Sample (n)** | **Key Themes** | **Key Findings** | **Relevance to Survey** |
| --- | --- | --- | --- | --- | --- |
| U.Rehman, 2022^2^ | Cross-sectional questionnaire study | 148 final-year medical students from two UK universities | Student and Trainee Perceptions | • 82% had no exposure; 48% interested in NSFA career  • >75% unaware of complications or training routes  • 68% believed one‑day course sufficient  • Calls to integrate NSFA into curricula for safety | • Supports items on interest vs. training gap  • Highlights knowledge deficits and regulation concerns  • Informs inclusion of modules on procedural overview and complication management |
| Clough, 2025^3^ | Cross-sectional questionnaire study | 142 UK plastic surgery trainees | Student and Trainee Perceptions | • 42% rated aesthetic training negatively  • Only 33% had dedicated aesthetic rotations  Aesthetic exposure largely dependent on unpaid private attendance  • 75% planned private aesthetic practice | • Highlights limited exposure and confidence deficits among trainees  • Informs survey domains on barriers, regulations and future training pathways. |
| Fell et al. 2020^4^ | National cross-sectional survey | 131 UK plastic surgery trainees | Student and Trainee Perceptions | • 82% respondents said that their deanery did not provide integrated aesthetic training  • Recommends that the Joint Committee on Surgical Training, Royal Surgical Colleges, Surgical Specialty Associations and independent sector work together to develop a model for training in aesthetic surgery | • Highlights limited exposure and confidence deficits among trainees  • Supports survey questions on independent aesthetics training pathways. |
| Grover, 2023^~~5~~^ | Cross-sectional survey | 59 Medical Students and 87 Dental students | Student and Trainee Perceptions, Informing Curricula and Training Pathways | • 89% had no NSFA teaching; none standardised nationally  • 66-75% support inclusion in curricula  • Minimal awareness of practice routes and ethics  • Highlights risk of non‑HCP providers for vulnerable patients | • Strongly supports items measuring lack of formal training and exposure  • Informs inclusion of curriculum content on procedures, complication management, and ethics |
| Paterson & Allison, 2006^6^ | Cross-sectional questionnaire study | 41 UK Specialist Registrars (SpRs) in Plastic Surgery | Student and Trainee Perceptions, Informing Curricula and Training Pathways | • 85% interested in aesthetics; only 10% felt prepared  • NHS cuts and “local guidelines” reduced training opportunities  • 83% lacked private clinic exposure  • Universal call for formal integration of aesthetic training | • Supports survey items on training disparity and preparedness  • Justifies inclusion of ethics and patient expectation management content |
| D.H. Awal, 2019^7^ | Cross-sectional survey | 45 OMFS Trainees | Student and Trainee Perceptions, Informing Curricula and Training Pathways, Barriers | • Large inter‑specialty variation; OMFS least comprehensive  • 80% of OMFS trainees report insufficient training  • 47% cite poor NHS access as barrier  • Advocates inclusion of non‑surgical modules | • Supports survey items on inadequate training access and barriers  • Justifies questions on formal, GMC‑regulated pathways  • Informs inclusion of complication‑management education |
| Baker & Winterton, 2022^8^ | Cross-sectional questionnaire and logbook database review | 285 plastic surgery registrars | Informing Curricula and Training Pathways | • 88% of aesthetic cases logged within NHS-unrepresentative mix  • Independent sector exposure limited to assisting (86%)  • Most private experience ad‑hoc and unstructured  • Calls for IS fellowships and mentorship schemes | • Supports items measuring inadequate independent sector exposure  • Justifies need for formal training pathway and regulation  • Informs survey on curriculum reforms for consent and patient‑management content |
| N.M. Pantelides, 2018^9^ | Retrospective analysis of national eLogbook data (2010–2016) | 454 plastic surgery registrars | Informing Curricula and Training Pathways | • Avg. 122 aesthetic ops/trainee; 50% only assistants  • Experience concentrated in breasts/body; minimal facial work  • Meets RCS criteria in only 2 of 8 domains  • Training restricted by NHS funding and hours | • Evidence for including items on narrow training scope and missing competencies  • Supports advocacy for fellowship pathways and curricular reform  • Informs survey questions on financial barriers to external courses |
| Whitaker et al., 2007^10^ | Retrospective audit of aesthetic procedures logged at a single UK plastic surgery unit | 99,651 total plastic surgery procedures; 1,361 were purely aesthetic | Informing Curricula and Training Pathways | • NHS aesthetic volume increasing (2000–2005); mainly breast cases  • Highlights perception of limited opportunities despite growth  • Endorses dedicated aesthetic rotation and private practice access | • Provides historical context for assessing current exposure in survey  • Supports inclusion of items evaluating sufficiency of training and need for dedicated modules |
| Chasapi & Salibi 2021^11^ | Cross-sectional survey | 83 BAAPS members | Informing Curricula and Training Pathways, Ethical Practice | • 74% value pre‑op screening; only 50% practise it  • 89% rely on intuition; 24% use validated tools  • 64% report no training; 78% want curriculum inclusion | • Supports items addressing untrained psychological screening  • Justifies inclusion of ethics and BDD assessment curriculum questions  • Informs survey on barriers from lack of formal training |
| Lee et al., 2017^12^ | Cross-sectional two-stage study assessing associations between bullying involvement and desire for cosmetic surgery | 752 adolescents (11-16) included (bullies, victims, bully-victims, uninvolved) | Ethical Practice | • Bullied adolescents show higher desire for cosmetic surgery  • Victims have lower self‑esteem and more emotional issues  • Bullies’ interest linked to social dominance  • Authors call for psychological screening in pre‑surgery assessment | • Evidence for including items addressing BDD and psychological screening  • Informs curriculum justification for teaching aesthetic patient psychology  • Supports inclusion of ethics and marketing influence questions |
| J.Olding, 2025^13^ | Cross-sectional logbook database review and content analysis | 339 GMC-registered aesthetic practitioners from the British College of Aesthetic Medicine (BCAM) register | Regulation, Ethical Practice | • 62% misused the title “surgeon”; many not specialist‑registered  • Some offered major surgery or non‑surgical work outside scope  • Legal use of “surgeon” conflicts with RCS/GMC/ASA guidance | • Justifies survey questions on regulatory confusion and role boundaries  • Supports inclusion of content on UK regulatory framework and marketing ethics |
| E. Gunn, 2014^14^ | Observational, cross-sectional study | Analysed the content of 125 websites against a set criteria based on existing advertising regulations | Media, Ethical Practice, Regulation | • 22% of 125 websites breached advertising rules  • 20 sites illegally promoted POMs; 50% lacked promo expiry dates  • 27% omitted practitioner qualifications  • Most non‑surgical providers were nurses/dentists  • 78% failed to mention complications | • Justifies items measuring concern over advertising ethics and regulation lapses  • Supports inclusion of business/marketing ethics in training  • Informs questions on who should perform aesthetic procedures and standards transparency |
| R. Nassab, 2010^15^ | Observational, cross-sectional study | 100 Websites, 50 from New York, 50 from London | Media, Regulation, Barriers | • 26% UK vs 12% US sites used unethical discounts/free treatments  • 36% lacked or misrepresented practitioner credentials  • <40% mentioned complications  • Internet advertising largely unregulated | • Supports items on marketing and advertising regulation concerns  • Informs need for teaching marketing ethics and consent  • Highlights lack of regulation and practitioner misrepresentation for survey items |
| C. Asher, 2019^16^ | Retrospective Study | 11 patients managed for complications from private cosmetic surgery in a single NHS trust between Jan 2016 to Mar 2017 | Media, Cosmetic Tourism, Regulation | • £259,732 cost to NHS from 11 cases  • Most expensive: patients treated abroad (Turkey, DR)  • Drivers: low cost, anonymity, weak regulation  • Limited post‑op access to overseas providers  • Surgery commodification linked to poor ethics and weak advertising laws | • Supports items measuring prevalence of cosmetic tourism complications among clinicians  • Justifies inclusion of questions on marketing ethics, commodification, and regulation  • Informs curriculum items on complication management and regulatory education |
| R. Jeevan, 2010^17^ | Cross-sectional survey | 203 UK consultant members of BAPRAS | Media, Cosmetic Tourism | • 37% of consultants managed NHS cases from surgery abroad  • 60% report growing frequency  • NHS provides emergency, not elective, revisions  • Informed consent often inadequate abroad  • “After‑care insurance” proposed as a solution | • Informs prevalence questions on managing tourism complications  • Underpins survey items on NHS policy, consent, and complication distinction teaching  • Supports curriculum inclusion on complication management and regulation |
| J. Wokes, 2022^18^ | Cross-sectional survey | 97 BAPRAS Members | Media | • 51% experienced online defamation; 78% pressured for refunds  • 32% reported mental health impact  • 67% felt unsupported by professional bodies  • Surgeons adopt defensive, selective practice | • Supports inclusion of items on stigma, workplace pressures, and ethical barriers  • Justifies teaching business and marketing ethics  • Informs curriculum on consent, expectations, and psychological screening |

# **Review Limitations**

Limitations of this scoping review include the restriction to English-language publications, which may have excluded relevant international perspectives. Additionally, the inclusion of grey literature was limited, potentially missing non-indexed educational reports or internal training documents.

# **References**

1. PRISMA. PRISMA for Scoping Reviews (PRISMA-ScR).<https://www.prisma-statement.org/scoping>. [Accessibility verified December 23, 2025]
2. Rehman U, Freer FAJ, Sarwar MS, et al. Non-surgical facial aesthetics: should this be incorporated into medical education? Adv Oral Maxillofac Surg 2022;8:100327. doi:10.1016/j.adoms.2022.100327
3. Clough R, Gavala MI, Rafie A, et al. Plastic surgery training in the United Kingdom and Ireland in 2025: results of the National Training Survey. J Plast Reconstr Aesthet Surg 2025. doi:10.1016/j.bjps.2025.09.031
4. Fell MJ, Staruch R, Baker B, et al. Plastic surgery training in the UK: results from a national survey of trainee experiences. JPRAS Open 2020;25:72–82. doi:10.1016/j.jpra.2020.06.003
5. Grover S, Patel M. Attitudes and perceptions of medical and dental students on the implementation of non-surgical facial aesthetics in their curricula. Br J Oral Maxillofac Surg 2023;61(10):e74–e81. doi:10.1016/j.bjoms.2023.07.015
6. Paterson P, Allison K. Maintaining standards of aesthetic practice in trainees subject to NHS restrictions. J Plast Reconstr Aesthet Surg 2006;59(8):856–859. doi:10.1016/j.bjps.2005.10.006
7. Awal DH, McArdle P, Parmar S. Review of the Intercollegiate Surgical Curriculum Programme for oral and maxillofacial surgery aesthetics: are we underselling ourselves? Br J Oral Maxillofac Surg 2019;57(10):1076–1082. doi:10.1016/j.bjoms.2019.08.011
8. Baker R, Winterton R. Optimising UK training in aesthetic surgery: a prospective national study. J Plast Reconstr Aesthet Surg 2022;75(12):4685–4695. doi:10.1016/j.bjps.2021.10.036
9. Pantelides NM, Griffiths D, Gault D. An analysis of the cosmetic surgery experience acquired through UK plastic surgery training. J Plast Reconstr Aesthet Surg 2018;71(7):951–959. doi:10.1016/j.bjps.2018.03.013
10. Whitaker IS, Karoo R, Shokrollahi K, et al. An analysis of 1361 aesthetic procedures from 2000 to 2005 in a large regional plastic surgery unit: implications for cosmetic surgery training. J Plast Reconstr Aesthet Surg 2007;60(9):1009–1014. doi:10.1016/j.bjps.2006.12.011
11. Chasapi M, Salibi A. The psychological assessment of aesthetic patients: results of a survey of BAAPS members. Aesthet Surg J 2021;41(6):NP706–NP714. doi:10.1093/asj/sjaa376
12. Lee HR, Chen W, Wong C, et al. Associations between bullying involvement and desire for cosmetic surgery: a cross-sectional two-stage study. Plast Reconstr Surg 2017;139(5):865e–873e. doi:10.1097/PRS.0000000000003207
13. Olding J, D’Souza A. Call yourself a surgeon? The use of the title surgeon among non-surgical cosmetic practitioners in the UK. JPRAS Open 2025;5(1):100103. doi:10.1016/j.jpra.2025.100103
14. Gunn E, Wainwright DJ. Cosmetic websites Scotland: legal or lurid. J Plast Reconstr Aesthet Surg 2014;67(8):1078–1084. doi:10.1016/j.bjps.2014.04.052
15. Nassab R. Online marketing strategies of plastic surgeons and clinics: UK vs USA. Aesthet Surg J 2011;31(5):566–571. doi:10.1177/1090820X11402456
16. Asher CM, Fleet M, Jivraj B, et al. Cosmetic tourism: a costly filler within the NHS budget or a missed financial opportunity? Aesthet Plast Surg 2020;44(2):586–594. doi:10.1007/s00266-019-01571-7
17. Jeevan R, Birch J, Armstrong AP. Travelling abroad for aesthetic surgery: informing healthcare practitioners and providers while improving patient safety. J Plast Reconstr Aesthet Surg 2011;64(2):143–147. doi:10.1016/j.bjps.2010.09.017
18. Wokes J, Wade RG. Fake news, defamation, online reviews, and their potential devastating consequences for aesthetic plastic surgeons. Aesthet Surg J 2022;42(8):NP546–NP552. doi:10.1093/asj/sjac08
