# Supplementary material for: SCULPT: Medical student and resident doctor comprehension, uptake of learning and perception of aesthetic surgery and training
Source: JPRAS Open. 2026 Apr 4;50:10–25. doi: 10.1016/j.jpra.2026.03.043 (PMC13127476; doi:10.1016/j.jpra.2026.03.043)
Supplement: Supplementary file 2 [file mmc2.docx]

Appendix B: Survey Tool

# Section 1: Participant Role

**1. Please select the option that best describes your current role:**

- ☐ Medical Student (currently enrolled at a UK medical school)
- ☐ Resident Doctor (currently in a UK training programme)

*(Based on the answer to Q1, the survey will branch to either Section 2A for Students or Section 2B for Doctors)*

# Section 2A: Medical Student Experience

*(This section is for Medical Students only)*

**1. What is your year of study?**

- ☐ Year 1 (Undergraduate)
- ☐ Year 2 (Undergraduate)
- ☐ Intercalating (Undergraduate)
- ☐ Year 3 (Undergraduate)
- ☐ Year 4 (Undergraduate)
- ☐ Year 1 (Graduate)
- ☐ Year 2 (Graduate)
- ☐ Intercalating (Graduate)
- ☐ Year 3 (Graduate)
- ☐ Year 4 (Graduate)

**2. What medical school do you attend?**

- Anglia Ruskin University
- Aston Medical School Bangor
- University Barts and The London School of Medicine and Dentistry
- Brighton and Sussex Medical School
- Bristol Medical School Brunel Medical School
- Buckingham University Medical School
- Cardiff University School of Medicine
- Edge Hill University Medical School
- Imperial College School of Medicine
- Keele University School of Medicine
- Kent and Medway Medical School
- King’s College London
- Lancaster Medical School
- Leeds School of Medicine
- Leicester Medical School
- Newcastle University Medical School
- Plymouth University
- Peninsula Medical School
- Queen’s University Belfast Medical School
- St George’s, University of London
- Swansea University Medical School
- Ulster University Medical School
- University College London Medical School
- University of Aberdeen School of Medicine
- University of Birmingham Medical School
- University of Cambridge School of Clinical Medicine
- University of Central Lancashire School of Medicine
- University of Dundee School of Medicine
- University of East Anglia (Norwich Medical School)
- University of Edinburgh Medical School
- University of Exeter Medical School
- University of Glasgow Medical School
- University of Hull York Medical School
- University of Lincoln Medical School
- University of Liverpool Medical School
- University of Manchester Medical School
- University of Nottingham Medical School
- University of Oxford Medical School
- University of Sheffield Medical School
- University of Southampton Medical School
- University of St Andrews School of Medicine
- University of Sunderland School of Medicine
- University of Warwick Medical School

**3. What is your gender?**

- ☐ Male
- ☐ Female
- ☐ Non-binary/third gender
- ☐ Prefer not to say

**4. What is your ethnicity?**

- ☐ White
- ☐ Black or Black British
- ☐ Asian or Asian British
- ☐ Mixed/Multiple ethnic groups
- ☐ Other please specify:__________
- ☐ Prefer not to say

**5. Have you had any prior exposure to aesthetic medicine or surgery? (Select all that apply)**

- ☐ No prior exposure
- ☐ Yes, in a clinical placement (Plastics, Dermatology, ENT, etc.)
- ☐ Yes, observed an aesthetic surgical procedure
- ☐ Yes, observed a non-surgical aesthetic procedure
- ☐ Yes, attended a workshop or conference on aesthetics
- ☐ Other (please specify): __________

**6. Have you received any formal teaching on aesthetic medicine or surgery in medical school?**

- ☐ Yes, as part of the core curriculum
- ☐ Yes, as an optional/elective module
- ☐ No

**7. If yes, what form did this teaching take? (Select all that apply)**

- ☐ Surgical aesthetics (e.g., facelifts, rhinoplasty)
- ☐ Non-surgical aesthetics (e.g., Botox, fillers)
- ☐ Lectures
- ☐ Workshops
- ☐ Clinical placements
- ☐ Other (please specify): __________

**8. Have you received any teaching on aesthetics outside of medical school? (e.g., student societies, private courses)**

- ☐ Yes
- ☐ No

**9. How accessible do you think aesthetic medicine and surgery training is for medical students? (1 = Not accessible at all, 5 = Very accessible)**

- ☐ 1 ☐ 2 ☐ 3 ☐ 4 ☐ 5

*(Medical students will now skip to Section 3)*

# Section 2B: Resident Doctor Experience

*(This section is for Resident Doctors only)*

**1. What is your current training grade?**

- ☐ Foundation Year 1 (FY1)
- ☐ Foundation Year 2 (FY2)
- ☐ Core Trainee (CT1/2/3)
- ☐ Specialty Trainee / Registrar (ST3+)
- ☐ GP Trainee (GPVTS)
- ☐ Other (please specify): __________

**2. If ST3 +, What is your primary specialty or training programme?**

- *(e.g., General Practice, Core Surgical Training, Acute Medicine)* __________

**3. In which UK region or deanery are you currently training?**

- East Midlands
- East of England
- Kent, Surrey & Sussex (KSS)
- London
- North East
- North West (Mersey & North West)
- Northern Ireland
- Scotland
- South West (Peninsula & Severn)
- Thames Valley
- Wales
- Wessex
- West Midlands
- Yorkshire and the Humber

**4. What is your gender?**

- ☐ Male
- ☐ Female
- ☐ Non-binary/third gender
- ☐ Prefer not to say

**5. What is your ethnicity?**

- ☐ White
- ☐ Black or Black British
- ☐ Asian or Asian British
- ☐ Mixed/Multiple ethnic groups
- ☐ Other please specify:__________
- ☐ Prefer not to say

**6. In medical school, did you receive any formal teaching on aesthetic medicine or surgery?**

- ☐ Yes
- ☐ No

**7. If yes, which form did this teaching take? (Select all that apply)**

- ☐ Surgical aesthetics (e.g. facelifts, rhinoplasty)
- ☐ Non-surgical aesthetics (e.g. Botox, fillers)
- ☐ Lectures
- ☐ Workshops
- ☐ Other please specify:__________

**8. Since graduating, have you undertaken any training/courses in aesthetics? (Select all that apply)**

- ☐ No postgraduate training in aesthetics
- ☐ Yes, a foundation course in Botulinum Toxin / Dermal Fillers
- ☐ Yes, an advanced course in Botulinum Toxin / Dermal Fillers
- ☐ Yes, a formal qualification (e.g., Level 7 Diploma)
- ☐ Yes, attended a conference or workshop on aesthetics
- ☐ Other (please specify): __________

**9. Have you ever managed a patient presenting with a complication from an aesthetic procedure?**

- ☐ Yes
- ☐ No

**10. If yes, were any of the complications you managed the result of aesthetic procedures performed outside the UK?**

- ☐ Yes
- ☐ No

**11. Have you ever assisted with or performed (under supervision) any aesthetic procedures?**

- ☐ Yes, a non-surgical procedure (e.g., injectables)
- ☐ Yes, a surgical procedure (e.g., pinnaplasty)
- ☐ No

*(Resident Doctors will now proceed to Section 3)*

# Section 3: Knowledge of Procedures & Complications

*(From section 3 onwards, All participants will answer these questions)*

**1. How would you rate your overall knowledge of aesthetic procedures? (1 = Not knowledgeable, 5 = Very knowledgeable)**

- Surgical aesthetic procedures ☐ 1 ☐ 2 ☐ 3 ☐ 4 ☐ 5
- Non-surgical aesthetic procedures ☐ 1 ☐ 2 ☐ 3 ☐ 4 ☐ 5

**2. How knowledgeable are you about the following surgical procedures? (1 = Not at all familiar, 5 = Very familiar)**

- Rhytidectomy (Facelift): ☐ 1 ☐ 2 ☐ 3 ☐ 4 ☐ 5
- Rhinoplasty: ☐ 1 ☐ 2 ☐ 3 ☐ 4 ☐ 5
- Pinnaplasty (Otoplasty): ☐ 1 ☐ 2 ☐ 3 ☐ 4 ☐ 5
- Browlift: ☐ 1 ☐ 2 ☐ 3 ☐ 4 ☐ 5
- Blepharoplasty: ☐ 1 ☐ 2 ☐ 3 ☐ 4 ☐ 5
- Lip lift: ☐ 1 ☐ 2 ☐ 3 ☐ 4 ☐ 5
- Genioplasty: ☐ 1 ☐ 2 ☐ 3 ☐ 4 ☐ 5
- Implants for Head & Neck: ☐ 1 ☐ 2 ☐ 3 ☐ 4 ☐ 5
- Liposuction: ☐ 1 ☐ 2 ☐ 3 ☐ 4 ☐ 5
- Lipofilling (Fat Transfer): ☐ 1 ☐ 2 ☐ 3 ☐ 4 ☐ 5
- Breast reduction: ☐ 1 ☐ 2 ☐ 3 ☐ 4 ☐ 5
- Breast augmentation: ☐ 1 ☐ 2 ☐ 3 ☐ 4 ☐ 5
- Breast lift (Mastopexy): ☐ 1 ☐ 2 ☐ 3 ☐ 4 ☐ 5
- Abdominoplasty: ☐ 1 ☐ 2 ☐ 3 ☐ 4 ☐ 5
- Brachioplasty: ☐ 1 ☐ 2 ☐ 3 ☐ 4 ☐ 5
- Thigh lift: ☐ 1 ☐ 2 ☐ 3 ☐ 4 ☐ 5
- Superficial gluteal lipofilling: ☐ 1 ☐ 2 ☐ 3 ☐ 4 ☐ 5
- Hair transplant: ☐ 1 ☐ 2 ☐ 3 ☐ 4 ☐ 5

**3. How knowledgeable are you about the following non-surgical procedures? (1 = Not at all familiar, 5 = Very familiar)**

- Botulinum toxin: ☐ 1 ☐ 2 ☐ 3 ☐ 4 ☐ 5
- Fillers: ☐ 1 ☐ 2 ☐ 3 ☐ 4 ☐ 5
- Chemical peel: ☐ 1 ☐ 2 ☐ 3 ☐ 4 ☐ 5
- Laser treatment: ☐ 1 ☐ 2 ☐ 3 ☐ 4 ☐ 5
- Microneedling: ☐ 1 ☐ 2 ☐ 3 ☐ 4 ☐ 5

**4. Which specialties do you believe commonly perform aesthetic procedures? (Select all that apply)**

- ☐ Plastic Surgery
- ☐ Dermatology
- ☐ ENT (Ear, Nose, and Throat)
- ☐ Maxillofacial Surgery
- ☐ Obstetrics and Gynaecology
- ☐ Ophthalmology
- ☐ General Practice
- ☐ Other (please specify): __________
- ☐ Not sure

**5. How technically demanding do you perceive aesthetic procedures to be? (1 = Minimal skill required, 5 = Highly complex & technical)**

- Surgical aesthetic procedures: ☐ 1 ☐ 2 ☐ 3 ☐ 4 ☐ 5
- Non-surgical aesthetic procedures: ☐ 1 ☐ 2 ☐ 3 ☐ 4 ☐ 5

**6. How strongly do you agree with the following statements? (1 = Strongly disagree, 5 = Strongly agree)**

- Complications in aesthetic procedures are more distressing to patients than complications in non-aesthetic procedures. ☐ 1 ☐ 2 ☐ 3 ☐ 4 ☐ 5
- Patients undergoing aesthetic procedures face greater societal judgment when complications occur. ☐ 1 ☐ 2 ☐ 3 ☐ 4 ☐ 5
- Psychological impacts of complications are more severe in aesthetic procedures than in non-aesthetic procedures. ☐ 1 ☐ 2 ☐ 3 ☐ 4 ☐ 5

# Section 4: Barriers, Ethics & Media

**1. To what extent do you agree with the following statements? (1 = Strongly disagree, 5 = Strongly agree)**

- Aesthetic surgery is an important field in medicine. ☐ 1 ☐ 2 ☐ 3 ☐ 4 ☐ 5
- Aesthetic surgery is a respected field within the medical community. ☐ 1 ☐ 2 ☐ 3 ☐ 4 ☐ 5
- Aesthetic surgery is a respected field amongst members of the wider public. ☐ 1 ☐ 2 ☐ 3 ☐ 4 ☐ 5

**2. To what extent do you agree with the following statements? (1 = Strongly disagree, 5 = Strongly agree)**

- Aesthetic surgery significantly improves patient quality of life. ☐ 1 ☐ 2 ☐ 3 ☐ 4 ☐ 5
- Aesthetic surgery is an essential part of plastic and reconstructive surgery. ☐ 1 ☐ 2 ☐ 3 ☐ 4 ☐ 5
- There is stigma around pursuing a career in aesthetic surgery ☐ 1 ☐ 2 ☐ 3 ☐ 4 ☐ 5
- The high financial cost of aesthetic surgery training influences its accessibility for trainees ☐ 1 ☐ 2 ☐ 3 ☐ 4 ☐ 5
- Aesthetic surgery is a profit-driven industry ☐ 1 ☐ 2 ☐ 3 ☐ 4 ☐ 5

**3. How significant are the following personal barriers to pursuing a career in aesthetic medicine or surgery (1 = Not at all significant, 5 = Very significant)**

- Lack of structured training ☐ 1 ☐ 2 ☐ 3 ☐ 4 ☐ 5
- Financial burden of training (e.g., private fellowships, courses) ☐ 1 ☐ 2 ☐ 3 ☐ 4 ☐ 5
- Lack of opportunities in medical school ☐ 1 ☐ 2 ☐ 3 ☐ 4 ☐ 5

**4. How significant are the following systemic/ cultural barriers to pursuing a career in aesthetic medicine or surgery (1 = Not at all significant, 5 = Very significant)**

- Ethical concerns (e.g. commercialization, unrealistic patient expectations) ☐ 1 ☐ 2 ☐ 3 ☐ 4 ☐ 5
- Competitive nature of the specialty ☐ 1 ☐ 2 ☐ 3 ☐ 4 ☐ 5
- Perceptions of the field in the general public ☐ 1 ☐ 2 ☐ 3 ☐ 4 ☐ 5
- Perceptions of the field in the medical community ☐ 1 ☐ 2 ☐ 3 ☐ 4 ☐ 5

**5. How concerned are you about the following ethical issues within the field of aesthetic surgery? (1 = Not at all concerned, 5 = Very concerned)**

- Managing patients with unrealistic expectations: ☐ 1 ☐ 2 ☐ 3 ☐ 4 ☐ 5
- Potential for treating patients with body dysmorphic disorder (BDD): ☐ 1 ☐ 2 ☐ 3 ☐ 4 ☐ 5
- Marketing and advertising practices used within the industry: ☐ 1 ☐ 2 ☐ 3 ☐ 4 ☐ 5
- “Over-treatment” or “upselling” of procedures: ☐ 1 ☐ 2 ☐ 3 ☐ 4 ☐ 5
- Lack of regulation in the non-surgical sector: ☐ 1 ☐ 2 ☐ 3 ☐ 4 ☐ 5
- The practice of trainees performing private aesthetic procedures alongside their formal medical training: ☐ 1 ☐ 2 ☐ 3 ☐ 4 ☐ 5

**6. How strongly have the following sources influenced your perception of aesthetics? (1 = Not at all strongly, 5 = Very strongly)**

- Social media (e.g., Instagram, TikTok) ☐ 1 ☐ 2 ☐ 3 ☐ 4 ☐ 5
- Television/Movies ☐ 1 ☐ 2 ☐ 3 ☐ 4 ☐ 5
- Medical school teaching ☐ 1 ☐ 2 ☐ 3 ☐ 4 ☐ 5
- Conferences and events ☐ 1 ☐ 2 ☐ 3 ☐ 4 ☐ 5
- Observations during clinical placements ☐ 1 ☐ 2 ☐ 3 ☐ 4 ☐ 5
- Personal experiences or family/friends ☐ 1 ☐ 2 ☐ 3 ☐ 4 ☐ 5

**7. How has your perception of aesthetics influenced your interest in pursuing the following careers? (1 = Decreased my interest, 2 = Neither increased nor decreased my interest, 3 = Increased my interest)**

- Plastic surgery ☐ 1 ☐ 2 ☐ 3
- Aesthetic surgery ☐ 1 ☐ 2 ☐ 3
- Aesthetic medicine ☐ 1 ☐ 2 ☐ 3

# Section 5: Career Interest & Training Pathways

**1. To what extent do you agree with the following statements? (1 = Strongly disagree, 5 = Strongly agree)**

- Aesthetic medicine and surgery are well integrated into medical school curriculum ☐ 1 ☐ 2 ☐ 3 ☐ 4 ☐ 5
- Aesthetic medicine and surgery should be taught formally at medical school ☐ 1 ☐ 2 ☐ 3 ☐ 4 ☐ 5
- Aesthetic medicine and surgery should have its own formal, GMC-regulated training pathway ☐ 1 ☐ 2 ☐ 3 ☐ 4 ☐ 5
- Aesthetic medicine and surgery should be formally recognised as specialties in their own right ☐ 1 ☐ 2 ☐ 3 ☐ 4 ☐ 5

**2. Do you think only medically trained doctors should be allowed to perform invasive non-surgical aesthetic procedures?**

- ☐ Yes
- ☐ No

**3. If no, which of the following professionals do you believe should be allowed to perform invasive non-surgical aesthetic procedures?**

- ☐ Doctors
- ☐ Dentists
- ☐ Nurses
- ☐ Pharmacists
- ☐ Allied healthcare professionals (e.g. physiotherapists, osteopaths, paramedics)
- ☐ Non-medical beauty practitioners
- ☐ Other (please specify): __________

**4.** **If non-medical beauty practitioners, do you have any suggestions of safeguards or regulations toensure patient safety? (Free text)**

- __________________________________________

**5. How interested are you in pursuing a career in aesthetic medicine or surgery? (1 = Not at all interested, 5 = Very interested)**

- ☐ 1 ☐ 2 ☐ 3 ☐ 4 ☐ 5

**6. If selected 3-5 for above, what are the primary factors? (Select all that apply)**

- ☐ Perceived work life balance
- ☐ Financial prospects
- ☐ Interest in technical artistic skill
- ☐ Ability to have a significant positive impact on patient confidence
- ☐ Entrepreneurial/Business opportunity
- ☐ Other (please specify): __________

**7. If you were to pursue a career involving aesthetic procedures, which specialty would you most likely do so through?**

- ☐ Plastic Surgery
- ☐ Dermatology
- ☐ ENT
- ☐ Maxillofacial Surgery
- ☐ Obstetrics and Gynaecology
- ☐ Ophthalmology
- ☐ General Practice
- ☐ Other (please specify): __________

**8. How strongly do you believe the following should be formally taught in the medical school curriculum? (1 = Strongly disagree, 5 = Strongly agree)**

- Principles of consent and managing patient expectations in elective settings: ☐ 1 ☐ 2 ☐ 3 ☐ 4 ☐ 5
- Overview of common surgical and non-surgical procedures: ☐ 1 ☐ 2 ☐ 3 ☐ 4 ☐ 5
- Assessment and management of complications of aesthetic procedures: ☐ 1 ☐ 2 ☐ 3 ☐ 4 ☐ 5
- Business and marketing ethics in medicine: ☐ 1 ☐ 2 ☐ 3 ☐ 4 ☐ 5
- Psychology of the aesthetic patient (including screening for BDD): ☐ 1 ☐ 2 ☐ 3 ☐ 4 ☐ 5
- The current regulatory landscape for aesthetics in the UK: ☐ 1 ☐ 2 ☐ 3 ☐ 4 ☐ 5
