# Supplementary material for: SCULPT: Medical student and resident doctor comprehension, uptake of learning and perception of aesthetic surgery and training
Source: JPRAS Open. 2026 Apr 4;50:10–25. doi: 10.1016/j.jpra.2026.03.043 (PMC13127476; doi:10.1016/j.jpra.2026.03.043)

# Supplementary Figure 1

**Regional distribution of survey respondents.**

(A) Choropleth map showing the distribution of medical student participants across UK medical school regions.
(B) Choropleth map showing the distribution of resident doctor participants across UK postgraduate training regions (deaneries).


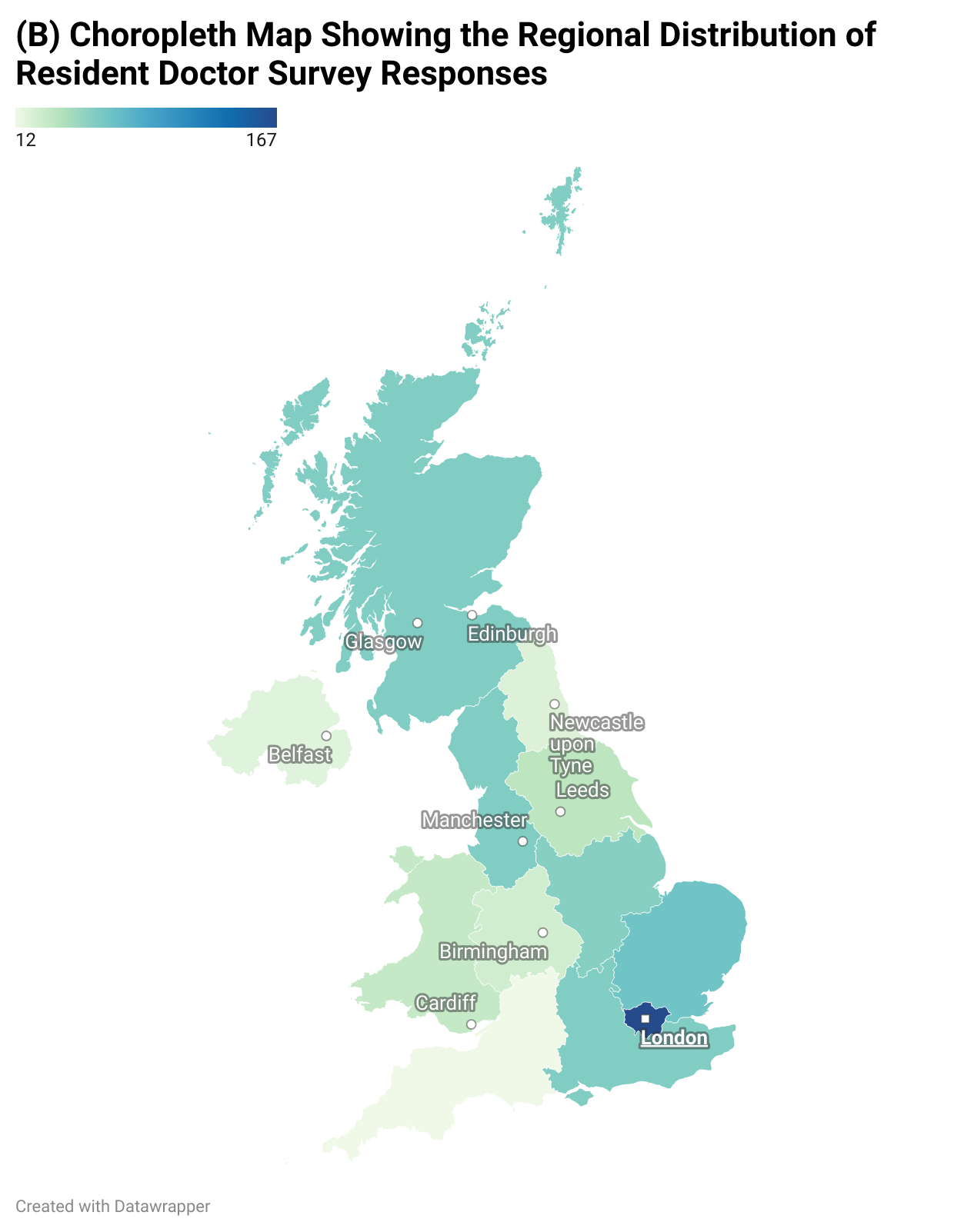


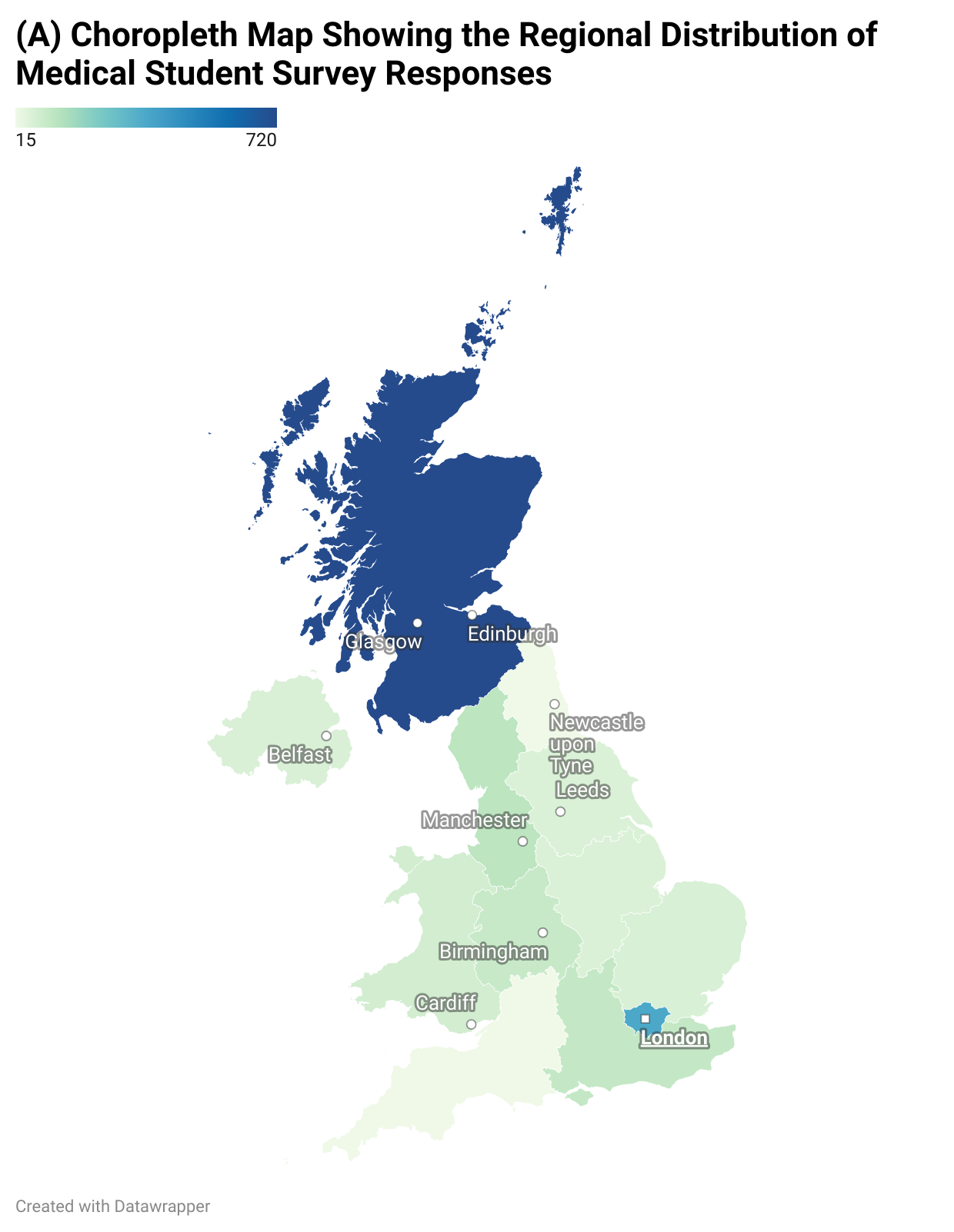

Supplement: Supplementary file 3 [file mmc3.docx]
