# Supplementary material for: SCULPT: Medical student and resident doctor comprehension, uptake of learning and perception of aesthetic surgery and training
Source: JPRAS Open. 2026 Apr 4;50:10–25. doi: 10.1016/j.jpra.2026.03.043 (PMC13127476; doi:10.1016/j.jpra.2026.03.043)

# Supplementary Figure 2

**Postgraduate aesthetic training among resident doctors**

Distribution of resident doctors (n = 73) reporting different forms of postgraduate aesthetic training, including foundation, advanced, and formal qualification-based pathways.


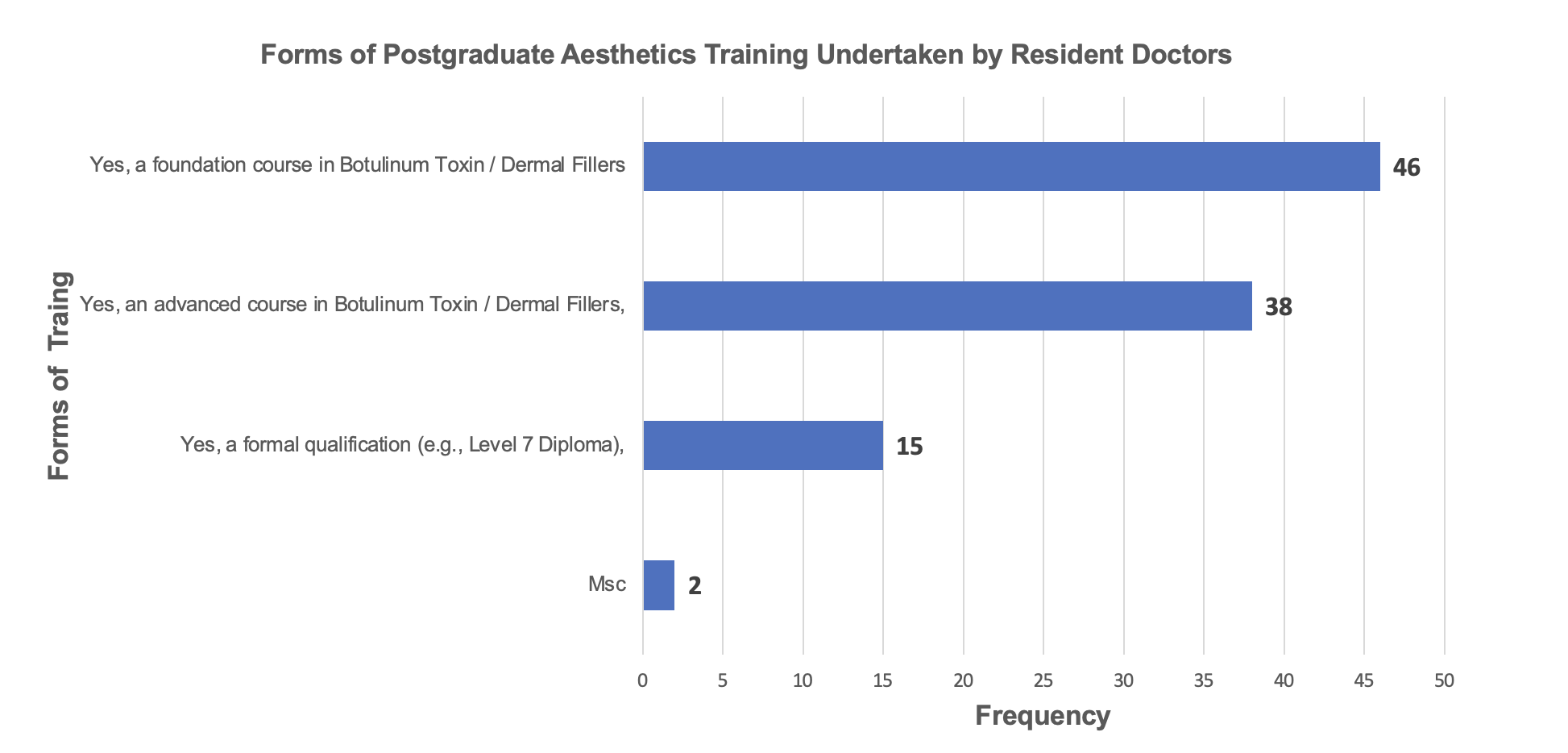

Supplement: Supplementary file 4 [file mmc4.docx]
