# Supplementary material for: SCULPT: Medical student and resident doctor comprehension, uptake of learning and perception of aesthetic surgery and training
Source: JPRAS Open. 2026 Apr 4;50:10–25. doi: 10.1016/j.jpra.2026.03.043 (PMC13127476; doi:10.1016/j.jpra.2026.03.043)

# Supplementary Figure 3

**Motivating Factors and levels of interest in aesthetic careers among Medical Students and Resident Doctors**

Interest in pursuing a career in aesthetics was expressed by 36.5% of students (n = 642) and 43.0% of doctors (n = 263). Among those expressing any level of interest (students: n = 1,060; doctors: n = 361), key motivating factors for students included the potential to positively impact patient confidence (n = 708) and interest in technical or artistic skill (n = 658). Resident doctors showed a similar pattern, with technical or artistic skill (n = 253) and financial prospects (n = 251) being the most common motivators.


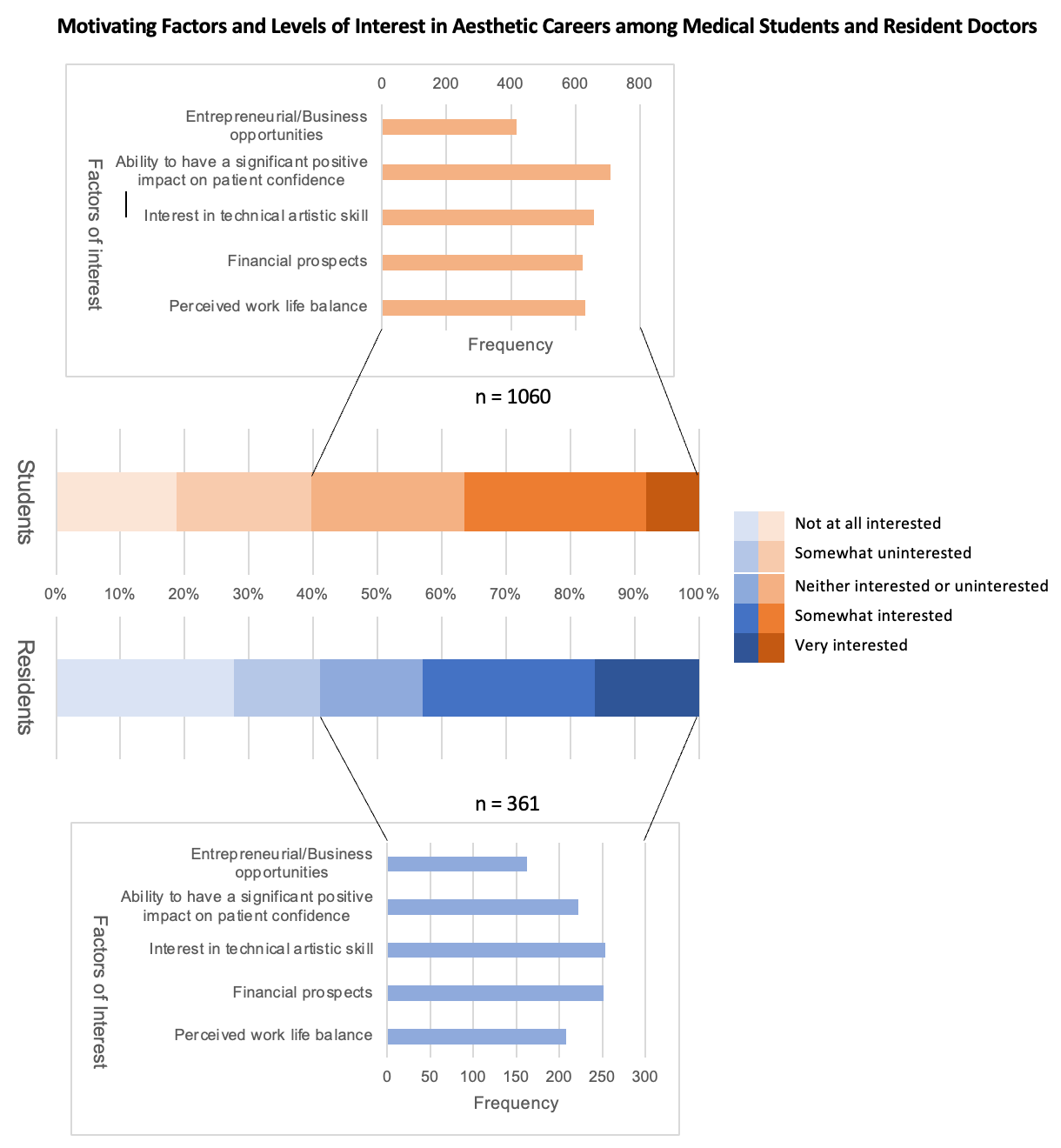

Supplement: Supplementary file 5 [file mmc5.docx]
