# Supplementary material for: SCULPT: Medical student and resident doctor comprehension, uptake of learning and perception of aesthetic surgery and training
Source: JPRAS Open. 2026 Apr 4;50:10–25. doi: 10.1016/j.jpra.2026.03.043 (PMC13127476; doi:10.1016/j.jpra.2026.03.043)
Supplement: Supplementary file 6 [file mmc6.docx]

# Supplementary Figure 4

**Participant agreement regarding the integration of aesthetics into undergraduate medical curricula**

Responses from medical students (N = 1,757) and resident doctors (N = 612) rating their agreement with statements on the extent to which aesthetic medicine and surgery are currently integrated into UK medical school curricula, and attitudes toward whether aesthetics should be formally taught. Ratings are presented on a five-point Likert scale.


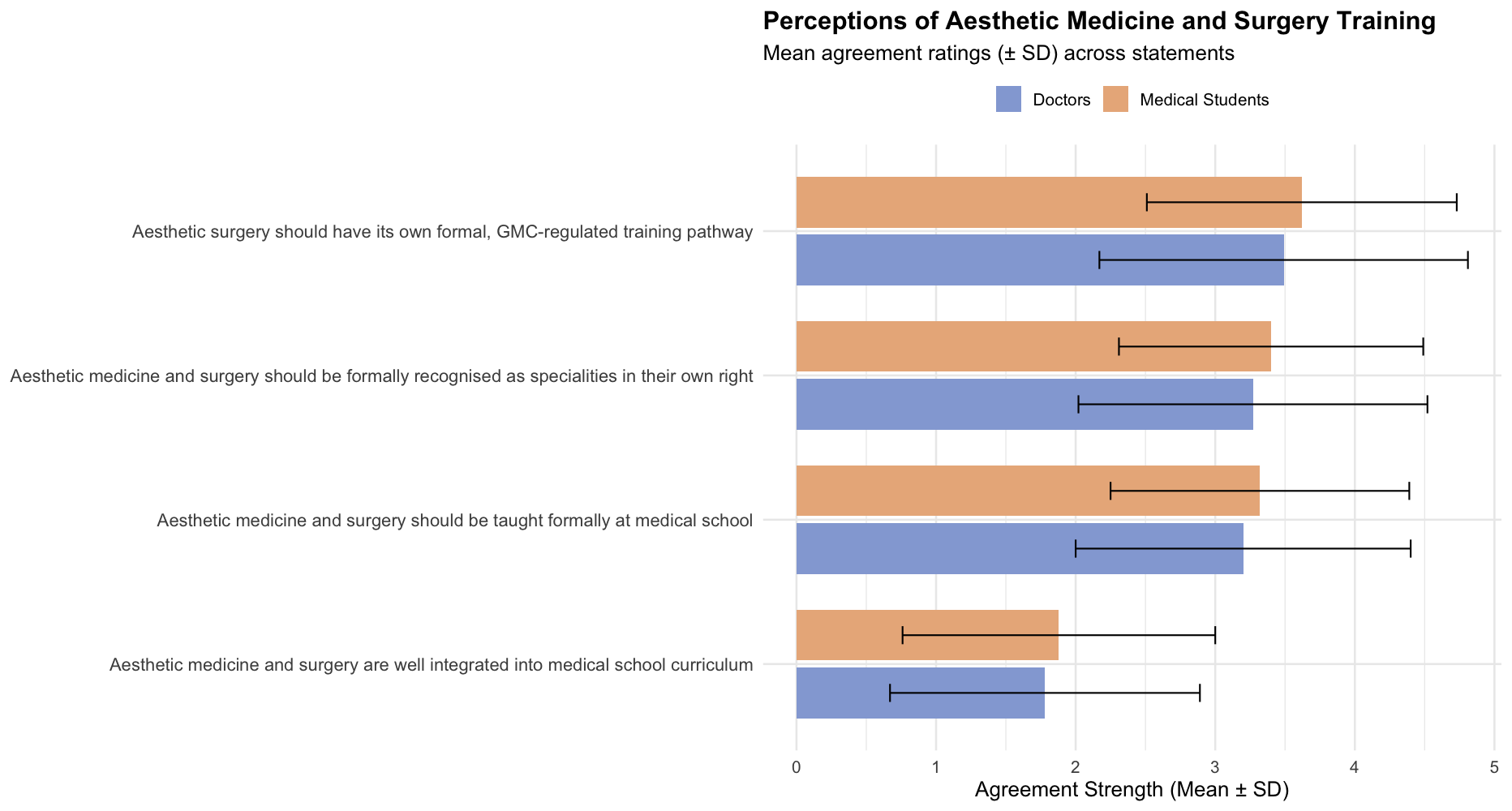


##

## 
