# Supplementary material for: SCULPT: Medical student and resident doctor comprehension, uptake of learning and perception of aesthetic surgery and training
Source: JPRAS Open. 2026 Apr 4;50:10–25. doi: 10.1016/j.jpra.2026.03.043 (PMC13127476; doi:10.1016/j.jpra.2026.03.043)
Supplement: Supplementary file 7 [file mmc7.docx]

# Supplementary Figure 5

**Perceived accessibility of aesthetic training**

Responses from medical students (N = 1757) rating accessibility of training opportunities on a five-point scale.


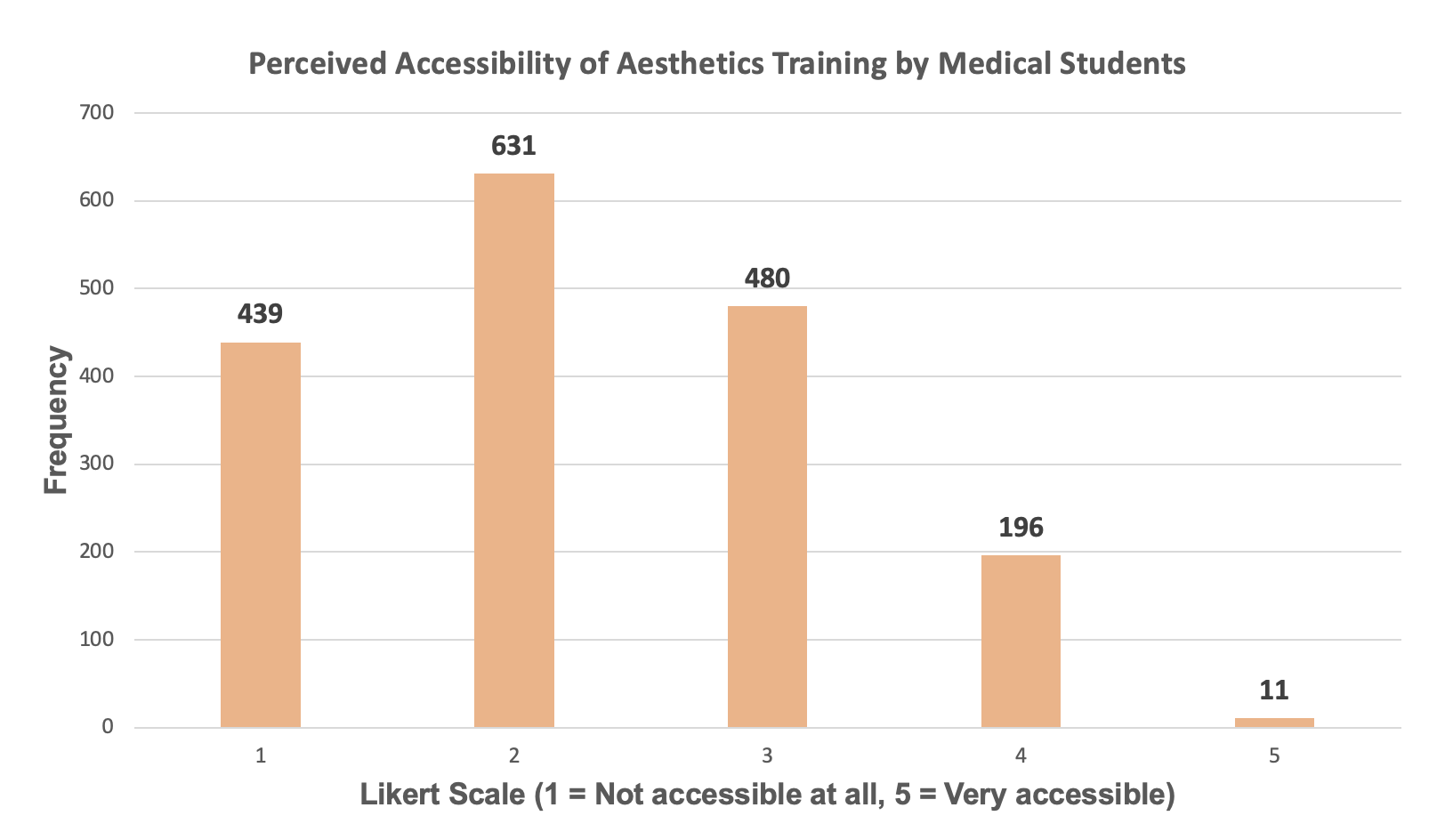


# 
