# Supplementary material for: SCULPT: Medical student and resident doctor comprehension, uptake of learning and perception of aesthetic surgery and training
Source: JPRAS Open. 2026 Apr 4;50:10–25. doi: 10.1016/j.jpra.2026.03.043 (PMC13127476; doi:10.1016/j.jpra.2026.03.043)
Supplement: Supplementary file 8 [file mmc8.docx]

# Supplementary Figure 6

**Perceived technical complexity of aesthetic procedures**

Mean ratings (± SD) from medical students and resident doctors describing perceived technical demand of surgical and non-surgical aesthetic procedures.


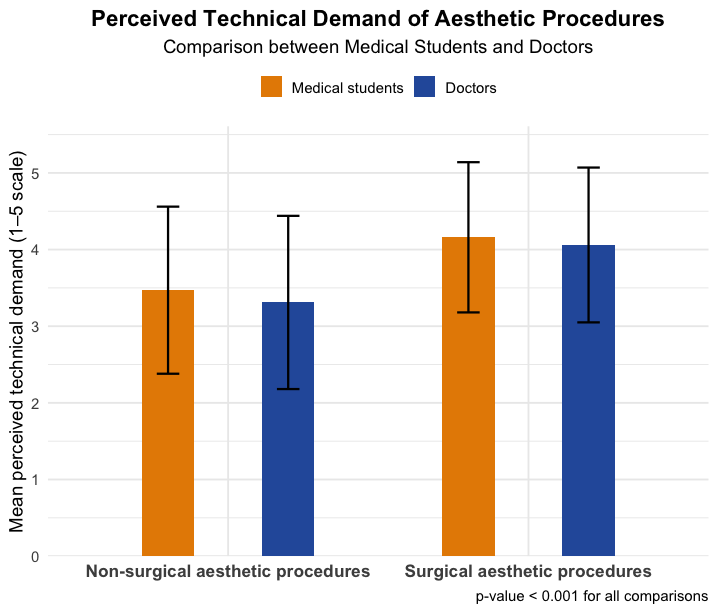


Medical students rated surgical aesthetic procedures as highly technically demanding (mean 4.16 ± 0.98) and non-surgical procedures as moderately demanding (3.47 ± 1.09). Resident doctors showed a similar pattern for both surgical (4.06 ± 1.01) and non-surgical procedures (3.31 ± 1.13).
