# Supplementary material for: SCULPT: Medical student and resident doctor comprehension, uptake of learning and perception of aesthetic surgery and training
Source: JPRAS Open. 2026 Apr 4;50:10–25. doi: 10.1016/j.jpra.2026.03.043 (PMC13127476; doi:10.1016/j.jpra.2026.03.043)

# Supplementary Figure 7

**Preferred specialty pathways for pursuing a career in aesthetic practice**

Distribution of medical students’ (N = 1,757) and resident doctors’ (N = 612) preferred specialty routes for entering aesthetic practice. Participants selected the specialty through which they would most likely pursue aesthetic surgery or aesthetic medicine (e.g., Plastic Surgery, Dermatology, ENT, Maxillofacial Surgery, General Practice, or other).


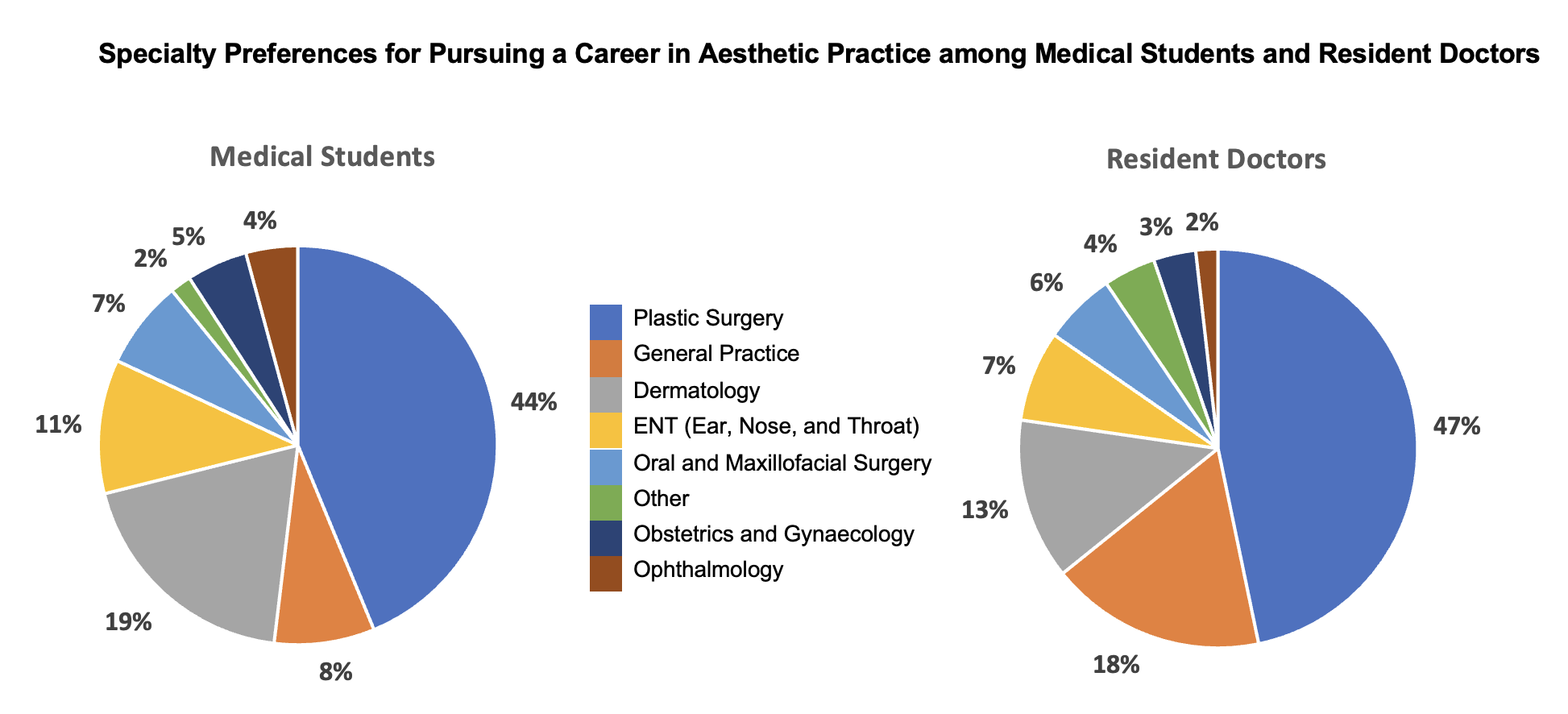

Supplement: Supplementary file 9 [file mmc9.docx]
