# Supplementary material for: SCULPT: Medical student and resident doctor comprehension, uptake of learning and perception of aesthetic surgery and training
Source: JPRAS Open. 2026 Apr 4;50:10–25. doi: 10.1016/j.jpra.2026.03.043 (PMC13127476; doi:10.1016/j.jpra.2026.03.043)
Supplement: Supplementary file 10 [file mmc10.docx]

# Supplementary Table 1

**Regional and institutional distribution of survey respondents.**Number of resident doctors and medical students by UK postgraduate training region (deanery) and medical school.

| **Region/Deanery** | **Resident Doctors (N = 612)** | **Medical School** | **Medical Students (N = 1757)** |
| --- | --- | --- | --- |
| East Midlands | 57 | Leicester Medical School | 28 |
|  |  | University of Nottingham | 27 |
|  |  | University of Lincoln Medical school | 3 |
| East of England | 67 | Anglia Ruskin University School of Medicine | 9 |
|  |  | University of Cambridge, School of Clinical Medicine | 34 |
|  |  | Norwich Medical School, University of East Anglia (UEA) | 20 |
| Kent, Surrey & Sussex | 43 | Brighton and Sussex Medical School | 4 |
|  |  | Kent and Medway Medical School | 5 |
| London | 167 | Barts and The London School of Medicine, Queen Mary University of London | 90 |
|  |  | Imperial College London | 117 |
|  |  | King’s College London GKT School of Medical Education | 41 |
|  |  | St George’s, University of London | 16 |
|  |  | UCL Medical School | 102 |
|  |  | Brunel Medical School | 3 |
| North East England | 21 | Newcastle University Medical School | 14 |
|  |  | Sunderland School of Medicine | 2 |
| North West England | 59 | Edge Hill University Medical School | 1 |
|  |  | Lancaster University Medical School | 60 |
|  |  | University of Liverpool School of Medicine | 21 |
|  |  | University of Manchester Medical School | 31 |
|  |  | University of Central Lancashire (UCLan) School of Medicine | 7 |
| Northern Ireland | 19 | Queen’s University Belfast School of Medicine | 44 |
|  |  | Ulster University Medical School | 18 |
| Scotland | 59 | University of Aberdeen School of Medicine | 348 |
|  |  | University of Dundee School of Medicine | 58 |
|  |  | University of Edinburgh Medical School | 216 |
|  |  | University of Glasgow School of Medicine | 85 |
|  |  | University of St Andrews School of Medicine | 13 |
| South West England | 12 | University of Bristol Medical School | 10 |
|  |  | University of Exeter Medical School | 2 |
|  |  | University of Plymouth, Peninsula Medical School | 3 |
| Thames Valley | 9 | University of Buckingham Medical School | 29 |
|  |  | University of Oxford Medical Sciences Division | 37 |
| Wales | 31 | Cardiff University School of Medicine | 44 |
|  |  | Swansea University Medical School | 31 |
|  |  | Bangor University | 2 |
| Wessex | 7 | Southampton Medical School | 30 |
| West Midlands | 26 | Aston University Medical School | 9 |
|  |  | University of Birmingham Medical School | 5 |
|  |  | Keele University School of Medicine | 52 |
|  |  | University of Warwick Medical School | 33 |
| Yorkshire and the Humber | 35 | Hull York Medical School | 29 |
|  |  | Leeds School of Medicine | 24 |
|  |  | Sheffield Medical School | 6 |
|  |  |  |  |
|  |  |  |  |
|  |  |  |  |
