# Supplementary material for: SCULPT: Medical student and resident doctor comprehension, uptake of learning and perception of aesthetic surgery and training
Source: JPRAS Open. 2026 Apr 4;50:10–25. doi: 10.1016/j.jpra.2026.03.043 (PMC13127476; doi:10.1016/j.jpra.2026.03.043)
Supplement: Supplementary file 11 [file mmc11.docx]

# Supplementary Table 2

**Knowledge scores for surgical and non-surgical aesthetic procedures**

Mean familiarity scores (± SD) for individual surgical and non-surgical procedures among medical students and resident doctors, rated on a five-point.

|  |  | **Resident Doctors Mean (SD)** | **Medical Students Mean (SD)** |
| --- | --- | --- | --- |
| **Surgical Procedures** | Rhytidectomy (Facelift) | 1.94 (1.22) | 2.09 (1.17) |
|  | Rhinoplasty | 2.37 (1.32) | 2.57 (1.25) |
|  | Pinnaplasty / Otoplasty | 2.08 (1.30) | 1.90 (1.10) |
|  | Browlift | 2.08 (1.28) | 2.04 (1.18) |
|  | Blepharoplasty | 2.17 (1.34) | 2.01 (1.17) |
|  | Lip lift | 2.01 (1.24) | 2.13 (1.20) |
|  | Genioplasty | 1.73 (1.12) | 1.72 (1.02) |
|  | Implants for head & neck | 1.76 (1.11) | 1.83 (1.08) |
|  | Liposuction | 2.54 (1.41) | 2.53 (1.25) |
|  | Lipofilling (Fat Transfer) | 2.28 (1.38) | 2.26 (1.25) |
|  | Breast reduction | 2.64 (1.40) | 2.49 (1.27) |
|  | Breast augmentation | 2.65 (1.39) | 2.58 (1.28) |
|  | Breast lift (Mastopexy) | 2.36 (1.39) | 2.20 (1.23) |
|  | Abdominoplasty | 2.41 (1.43) | 2.04 (1.19) |
|  | Brachioplasty | 1.76 (1.09) | 1.76 (1.05) |
|  | Thigh lift | 1.73 (1.11) | 1.80 (1.07) |
|  | Superficial gluteal lipofilling | 1.77 (1.10) | 1.75 (1.04) |
|  | Hair transplant | 2.43 (1.35) | 2.42 (1.29) |
|  | *Overall* | *2.36 (1.26)* | *2.24 (1.12)* |
| **Non-Surgical Procedures** | Botulinum toxin | 2.99 (1.37) | 2.82 (1.32) |
|  | Dermal fillers | 2.79 (1.38) | 2.73 (1.33) |
|  | Chemical peel | 2.32 (1.33) | 2.37 (1.32) |
|  | Laser treatment | 2.60 (1.35) | 2.58 (1.34) |
|  | Microneedling | 2.50 (1.38) | 2.55 (1.38) |
|  | *Overall* | *2.41 (1.28)* | *2.37 (1.19)* |

## 
